# Effects of resveratrol, curcumin, berberine and other nutraceuticals on aging, cancer development, cancer stem cells and microRNAs

**DOI:** 10.18632/aging.101250

**Published:** 2017-02-12

**Authors:** James A. McCubrey, Kvin Lertpiriyapong, Linda S. Steelman, Steve L. Abrams, Li V. Yang, Ramiro M. Murata, Pedro L. Rosalen, Aurora Scalisi, Luca M. Neri, Lucio Cocco, Stefano Ratti, Alberto M. Martelli, Piotr Laidler, Joanna Dulińska-Litewka, Dariusz Rakus, Agnieszka Gizak, Paolo Lombardi, Ferdinando Nicoletti, Saverio Candido, Massimo Libra, Giuseppe Montalto, Melchiorre Cervello

**Affiliations:** ^1^ Department of Microbiology and Immunology, Brody School of Medicine at East Carolina University, Greenville, NC 27858, USA; ^2^ Department of Comparative Medicine, Brody School of Medicine at East Carolina University, Greenville, NC 27858, USA; ^3^ Department of Internal Medicine, Hematology/Oncology Section, Brody School of Medicine at East Carolina University, Greenville, NC 27858, USA; ^4^ Department of Foundational Sciences, School of Dental Medicine, East Carolina University, Greenville, NC 27834, USA; ^5^ Department of Physiological Sciences, Piracicaba Dental School, State University of Campinas, Piracicaba, Brazil; ^6^ Unit of Oncologic Diseases, ASP-Catania, Catania 95100, Italy; ^7^ Department of Morphology, Surgery and Experimental Medicine, University of Ferrara, Ferrara, Italy; ^8^ Dipartimento di Scienze Biomediche e Neuromotorie, Università di Bologna, Bologna, Italy; ^9^ Chair of Medical Biochemistry, Jagiellonian University Medical College, Kraków, Poland; ^10^ Department of Molecular Physiology and Neurobiology, Wroclaw University, Wroclaw, Poland; ^11^ Naxospharma, Novate Milanese 20026, Italy; ^12^ Department of Biomedical and Biotechnological Sciences, Oncological, Clinical and General Pathology Section, University of Catania, Catania, Italy; ^13^ Biomedical Department of Internal Medicine and Specialties, University of Palermo, Palermo, Italy; ^14^ Consiglio Nazionale delle Ricerche, Istituto di Biomedicina e Immunologia Molecolare “Alberto Monroy”, Palermo, Italy

**Keywords:** miRs, SIRT, gene methylation, CSCs, natural products, resveratrol, curcumin

## Abstract

Natural products or nutraceuticals have been shown to elicit anti-aging, anti-cancer and other health-enhancing effects. A key target of the effects of natural products may be the regulation of microRNA (miR) expression which results in cell death or prevents aging, diabetes, cardiovascular and other diseases. This review will focus on a few natural products, especially on resveratrol (RES), curcumin (CUR) and berberine (BBR). RES is obtained from the skins of grapes and other fruits and berries. RES may extend human lifespan by activating the sirtuins and SIRT1 molecules. CUR is isolated from the root of turmeric (*Curcuma longa*). CUR is currently used in the treatment of many disorders, especially in those involving an inflammatory process. CUR and modified derivatives have been shown to have potent anti-cancer effects, especially on cancer stem cells (CSC). BBR is also isolated from various plants (*e.g., Coptis chinensis*) and has been used for centuries in traditional medicine to treat diseases such as adult- onset diabetes. Understanding the benefits of these and other nutraceuticals may result in approaches to improve human health.

## INTRODUCTION

Hippocrates is reported to have stated, “Let food be thy medicine and medicine be thy food”. Numerous natural products/nutraceuticals derived from food, plants and other organisms have been evaluated for their effects on various medical conditions including: inflammation, bacterial and viral infections, obesity, diabetes, mental disorders, cardiovascular problems and more recently cancer.

RES is being examined in at least 110 clinical trials on: age-related macular degeneration, aging, Alzheimer’s disease (AD), cancer including, (colon, follicular lymphoma, liver cancer, multiple myeloma (MM) and neuroendocrine tumors), cardiovascular problems including, (diastolic heart failure, hypertensive heart disease, cerebral blood flow, heart failure, heart failure with preserved ejection fraction), chronic renal insufficiency, cellulite, cognitive disorders, dyslipidemia, diabetes, diabetic nephropathy, endometriosis, eye diseases, Friedreich Ataxia, Huntington disease, memory, metabolic disorders, mood disorders, polycystic ovary syndrome (PCOS), seasonal allergic rhinitis, nonalcoholic fatty liver disease, peripheral arterial disease, obesity, schizophrenia, sedentary lifestyle, sports concussion, and other disorders.

CUR is being evaluated in at least 129 clinical trials for various conditions including: acute kidney injury, Alzheimer’s disease, atopic asthma, cancer (breast, head and neck cancer, osteosarcoma, chronic lymphocytic leukemia, glioblastoma, cutaneous T-cell lymphoma, colo-rectal and rectal cancer (CRC), non-small cell lung cancer, prostate, pancreatic neoplasms, multiple myeloma, myelodysplastic syndrome, cervical intraepithelial neoplasia), schizophrenia, cardiovascular abnormalities, chemotherapy induced oral mucositis, Crohn’s disease, chronic kidney disease, chronic obstructive pulmonary disease, chronic periodontitis, cystic fibrosis, diabetes (glucose tolerance and insulin resistance), end stage renal failure, erectile dysfunction, *Helicobacter Pylori* infection, hyperprolactinoma, inflammation in cancer patients, irritable bowel syndrome, knee osteoarthritis, lichen planus, major depression, metabolic syndrome, migraine, multiple sclerosis, optic atrophy, oral submucous fibrosis, proteinuria, psoriasis, radiation dermatitis, rheumatoid arthritis, ulcerative colitis, vascular stiffness and other health problems.

BBR is being examined in at least 35 clinical trials for treatment of: cardiovascular diseases, colorectal adenoma reoccurrence, diabetes, defective endothelial function, glucose metabolism/metabolic syndrome, *Helicobacter pylori* infection, hyperglycemia/glycemic control, hyper-lipemia, insulin sensitivity and insulin secretion, inflammation, non-alcoholic fatty liver disease, platelet aggregation, polycystic ovary syndrome and schizo-phrenia. Clearly these nutraceuticals, as well as many others, are being evaluated in the treatment of many health problems.

### Nutraceuticals effect on miR expression

Nutraceuticals (natural products) have been proposed to exert their effects on CSCs by interacting with the expression of miRs. miRs such as: miR-21, miR-22, miR-34, miR-101, miR-146a miR-200 and let-7 have been associated with the CSC phenotype. miRs may be involved in drug resistance, invasion, metastasis and angiogenesis, key events in the biological characteristics of CSCs [1, 2].

Natural products such as CUR, 3,3′-diindolylmethane, (-)-epigallocatechin-3-gallate, indole-3-carbinol, isoflavone, RES and others may alter the expression of miRs. These changes may result in suppression of cell growth and induction of apoptosis. The natural products may also alter epithelial to mesenchymal transition (EMT) and the sensitivity to chemotherapy [3].

### Resveratrol-an anti-aging, anti-cancer nutraceutical

RES has been evaluated in over 110 clinical trials, frequently consisting of diabetes and metabolic syndrome patients and certain types of cancer patients. In 2012, the global market for RES was estimated at $50 million (http://www.nutraingredients.com/Markets-and-Trends/US-dominates-global-resveratrol-market). Many different effects on cellular physiology have been attributed/proposed for RES. It has been reported that RES affects NF-kappaB activity and inhibits cytochrome P450 isoenzyme (CYP A1) drug metabolism and cyclooxygenase activity. Moreover, RES may influence TP53, FAS/FAS-ligand (FAS-L = CD95, tumor necrosis factor receptor superfamily member 6 [TNFRSF6]) induced apoptosis and mammalian target of rapamycin/mechanistic target of rapamycin (mTOR) activity and other biochemical pathways related to metabolism, oxidation of fatty acids, mitochondrial biogenesis and respiration and gluconeogenesis. RES may also induce apoptosis of activated T cells and suppress tumor necrosis factor-alpha (TNF-alpha), interleukin 17 (IL-17) and additional pro-inflammatory cytokines. Hence, it has been proposed that RES may be useful in auto-immune diseases. Importantly, RES may inhibit also the expression of hypoxia-inducible factor-1alpha (HIF-1alpha) and vascular endothelial growth factor (VEGF) and thus may have anti-cancer properties. RES may also affect brain-derived neurotropic factor expression which is important in obesity, diabetes, metabolic syndrome, depression, schizophrenia, bipolar disorder, and autism. Thus, RES may be an important natural product which could be used to treat many medical disorders [4].

RES is also present in *Polygonum cuspidatum*, which is also called bamboo in certain parts of the world and is considered an invasive weed. Thus, it could be inexpensive to produce large quantities of RES [5].

There are other compounds that are structurally related to RES that inhibit CSCs. Bedaquiline is an anti-microbiological agent that is used to treat multi-drug resistant tuberculosis. Recently it has been shown that bedaquiline inhibits the growth of mammary CSCs. Bedaquiline and RES inhibit the mitochondrial ATP-synthase. Both bedaquiline and RES also have anti-aging properties [6].

RES has been shown to inhibit pancreatic beta cell dysfunction, arterial stiffening and metabolic decline that can occur after administration of a high-fat/high-sugar (HFS) diet to nonhuman primates. In additional studies with this model, the effects on neuroprotection in the cortical brain tissue of these animals were investigated. These studies indicated that the non-human primates which had been fed RES and the HFS diet had neuroprotection against cerebral vascular dysfunction. Decreased mitochondrial aldehyde dehydrogenase 2 levels, dysregulation in endothelial nitric oxide synthase, and reduced capillary density were observed in the non-human primates fed RES and HFS diet in comparison to monkeys fed only the HFS diet [7].

### Effects of resveratrol on sirtuins in cancer and aging

RES induces sirtuins, a class of proteins involved in regulation of gene expression. Most sirtuins are histone deacetylates and in some cases, they are ADP-ribosyl transferases. The induction of sirtuins by RES may be responsible in part for the beneficial effects of the Mediterranean diet which is rich in RES [8].

The inherent defense mechanisms in plants results in the production of the phytoalexins RES and pterostilbene. These compounds can affect gene methylation, however, the concentrations required to elicit these changes are higher than normally obtained by casual consumption of food and beverage products containing these compounds. The effects of these compounds have been examined on the hepatocellular carcinoma HCC1806 cells and the triple negative breast cancer (TNBC) MDA-MB-157 cell line and as a control the non-malignant breast epithelial MCF-10A cells. Combination of RES and pterostilbene at physiologically relevant concentrations resulted in a synergistic inhibition of cell proliferation. The expression of the SIRT1, gamma-H2A histone family-member X (gamma-H2AX) and telomerase was detected at decreased levels in response to the combined treatment in HCC1806 and MDA-MB-157 cells but not in breast epithelial MCF-10A cells. Knockdown of SIRT1 mimicked the effects of resveratrol and pterostilbene on inhibition of SIRT1 in terms of the effects on telomerase and gamma-H2AX expression in HCC1806 breast cancer cells. The effects of these compounds on the ability of SIRT1 to recruit the DNA methyltransferases (DNMTs) were determined. These compounds resulted in down-regulation of the DNMT enzymes in the breast cancer cells but not in the MCF-10A cells, thus RES appeared to target SIRT1 specifically in breast cancer but not in normal cells [9].

RES is also considered to be a SIRT1-activating compound (STACs). STACs may have potential in cancer treatment. However, the effects of RES remain controversial as it has been reported to increase as well as decrease the effects of chemotherapy. The effects of RES and the synthetic STAC SRT1720 on etoposide and vincristine-induced cell death of Ewing’s sarcoma cells were examined. The effects of STACs can depend on the *TP53* gene status. In this study, the effects of STACs were examined in cells with different TP53 genotypes, Ewing’s sarcoma cells with WT (WE-68), mutant *TP53* (SK-ES-1) and *TP53* null (SK-N-MC) gene configurations. Interestingly, when used as a single agent, the effects of the STACs were independent of *TP53* gene status. However, when either SRT1720 or RES were added in combination with a chemotherapeutic drug, SRT1720 enhanced the effects of chemotherapeutic drugs on cell death while RES suppressed it [10].

The Klotho gene is a tumor suppressor gene that may be important in age-associated diseases. It is activated by RES in kidney cells. RES activates the activating transcription factor 3 (ATF-3)/c-Jun complex that results in Klotho expression. Dominant negative (DN) ATF-3 or c-Jun complexes suppressed the induction of Klotho in response to RES treatment [11].

In the *Invecchiare in Chianti* (InChianti) study, RES levels (metabolites in 24-hour urine samples) were not associated with improvement in the longevity of the patient populations (783 men and women, 65 years or older) or inflammation, cancer or cardiovascular disease. The authors in this study concluded that RES levels were not associated with the levels of serum C-reactive protein (CRP), interleukin-6 (IL-6), IL-1beta, TNF-alpha, cardiovascular disease, or cancer [12]. Thus, the roles of RES in anti-aging remain controversial.

### Effects of resveratrol on 5′ adenosine monophosphate-activated protein kinase (AMPK)

RES may target nutrient sensing pathways which are implicated in aging and cancer. These pathways include, among others, insulin/insulin like growth factor-1 (IGF-1), mTORC, AMPK and sirtuins. When these pathways are active, they affect the conversion of normal cells into senescent cells and can result in abnormal aging, AD and growth (cancer) [13, 14].

Caloric restriction (CR) will function as a neuroprotector in Apolipoprotein E (ApoE)-deficient mice. This has recently been shown to be due to the upregulation of fibroblast growth factor 21 (FGF21) expression which phosphorylates AMPK that leads to decreased mTOR signaling which is important in AD [15]. AMPK has also been shown to be important in the life span of *Caenorhabditis elgans*. Induction of AMPK was determined to increase life span [16].

The Sirtuin Sirt4 has been shown to regulate ATP homeostasis. Part of the effects of Sirt4 are due to a feedback loop involving AMPK [17].

RES has been shown to regulate TP53 expression. TP53 can regulate glucose metabolism. Treatment of serum-deprived or RES-treated cells resulted in increased levels of the tumor suppressor TP53 protein. RES was shown to increase the TP53 promoter activity in HeLa S3 cells. This has been suggested to be in part responsible for the anti-aging effects that are elicited by RES [18].

### Resveratrol and senescence

The induction of premature senescence after DNA damage in human primary dermal fibroblasts (BJ) has been associated with down-regulation of SIRT1 and SIRT2. RES was shown to decrease BJ proliferation and induce premature senescence. The phosphorylation of gamma-H2AX, and increased levels of TP53, p21^Cip-1^ and p16^INK4A^ were detected after RES treatment of BJ cells. In contrast, decreased levels of SIRT1 and SIRT2 were observed after treatment of BJ cells with concentrations of RES which induced premature cellular senescence. Silencing SIRT1 or SIRT2 or treatment with sirtinol induced premature cellular senescence. Doxorubicin also suppressed SIRT1 and SIRT2 levels and induced premature senescence [19].

Decreased levels of miR-15b were associated with increased expression of SIRT4 in cellular senescence and in photoaged skin. SIRT4 is linked with regulation of life span, metabolism and mitochondrial dysfunction. Senescence triggered by UVB or gamma irradiation of human dermal fibroblasts resulted in increased SIRT4 expression. In contrast, decreased levels of miR-15b were detected. miR-15b can target the SIRT4 gene. These studies point to the importance of miR-15b in regulation of SIRT4 which is involved in cellular senescence, mitochondrial dysfunction and photoaging of human skin [20].

### Resveratrol and suppression of drug-induced cardiotoxicity

RES has been shown to suppress doxorubicin-induced cardiotoxicity. The effect of RES on doxorubicin-induced endoplasmic reticulum (ER) stress and cardiomyocyte apoptosis in H9c2 rat heart tissue cells was examined by determining the extent of activation of the SIRT1 pathway. Treatment of H9c2 cells with doxorubicin was associated with increased expression of glucose-regulated protein 78 (GRP78) and C/EBP homologous protein (CHOP). RES could maintain viability of the cells and decrease the expression of the ER stress proteins in the presence of doxorubicin. Combined treatment of RES and doxorubicin resulted in a significant increase in SIRT1 expression, whereas single treatment with either RES or doxorubicin only led to a slight increase in SIRT1 expression. Nicotinamide, a SIRT1 inhibitor, prevented the effects of RES on SIRT1 expression in doxorubicin-treated cells and suppressed the effects on GRP78 and CHOP. These results indicate that RES has protective effects on H9c2 cells on doxorubicin-induced ER stress through activation of the SIRT1 pathway which may be important in the survival of cardiac and other cell types [21].

In addition, RES has been shown to protect against doxorubicin-induced cardiotoxicity via restoration of SIRT1 and suppression of catabolic/apoptotic pathways controlled by ubiquitin specific peptidase 7 (USP7), a TP53-deubiquitinating protein. 2-month-old (young) and 8-month-old (old) senescence-accelerated mice prone 8 (SAMP8) were treated with doxorubicin, and doxorubicin and RES, in the presence or absence of SIRT1 inhibitors, sirtinol or EX527. RES suppressed the induction of cardiotoxicity induced by doxorubicin. In these mice, the SIRT1 inhibitors prevented these effects of RES on doxorubicin-induced damage [22].

### Resveratrol and exercise

Exercise can prevent some of the frailty which results from aging. The effects of RES supplementation to aged and young mice (C57BL/6J, 16 month-old and 10 week-old, respectively) that were involved in an exercise regime were analyzed. The performance of the mice was evaluated using forelimb grip strength and exhaustive swimming exercise. The plasma levels of lactate, ammonia, glucose, and creatine kinase were evaluated after exercise. The addition of RES improved the performance of the old but not young mice. The combination of RES and exercise training together was necessary to achieve enhanced performance and it may limit the extent of deterioration that normally occurs with aging [23].

The heart function in old rats has been shown to improve upon exercise or treatment with RES. While the RES and exercise treatments each resulted in activation of the PI3K/PTEN/Akt pathway, only the RES-treated rats displayed elevated SIRT1. The combination of RES and exercise treatment was determined to enhance FOXO3 phosphorylation by activation of PI3K/PTEN/Akt and SIRT1 pathways in the swimming exercise model of aged rats. Increased levels of SIRT1 and PI3K/PTEN/Akt were detected in the groups of rats that underwent treatment with RES and exercise treatment as well as increased levels of phosphorylated FOXO3 which results in its inactivation [24].

The effects of exercise training and RES on vascular health in aging is influenced by various factors. Impaired vascular function is often due to endothelial dysfunction. This may be mediated by an altered redox balance which may result from increased reactive oxygen species (ROS) and decreased antioxidant ability. These changes result in decreased amounts of nitric oxide (NO). Physical inactivity and aging have negative effects on the vascular system. The NO bioavailability, redox balance and plasma lipids are improved by exercise. Some of these effects are mediated by SIRT1, AMPK and the estrogen receptor (ER). It turns out that RES can also activate these pathways, however, in some cases, RES can counteractive the positive effects of regular exercise [25].

Resveratrol enriched rice (DJ526) has been generated by genetic modifications that enables the rice plant to synthesize RES. This rice has been examined for its effects on physical strength and motor coordination. This DJ526 rice improved changes associated with aging [26].

### Resveratrol and diabetes

RES induces SIRT1, that may modulate Foxo1 signaling which is important in insulin signaling. Foxo1 inhibits glucose uptake and utilization in skeletal muscles. SIRT1 may deacetylase and suppress Foxo-1 which has effects on the transactivation of pyruvate dehydrogenase lipoamide kinase 4 (PDK4). PDK4 is a negative regulator of the glycolytic enzyme pyruvate dehydrogenase (PDH). Thus, RES has effects on glycolysis in aging skeletal muscle and insulin sensitivity. RES may have effects on prevention of reduction of skeletal muscle and insulin sensitivity, two important components in aging and diabetes [27].

Many of the beneficial effect of RES, CUR and BBR have been attributed to their anti-inflammatory properties. They have beneficial effects in pancreatic beta-cell function by suppressing phosphodiesterase activity which plays critical roles in glucose- and incretin-stimulated insulin secretion that is important in type 2 diabetes. The phosphodiesterases, which normally degrade cAMP, are important targets in type 2 diabetes. Recently, it was determined that both RES and CUR inhibit phosphodiesterase activity. RES and CUR were observed to suppress the expression of mRNAs encoding many of the phosphodiesterase isoforms such as: PDE3B, PDE8A, and PDE10A. These phosphodiesterase isoforms are important in the regulation of insulin secretion in the islets. These results suggest that RES and CUR could be important in the treatment of certain diabetics as they may enhance pancreatic beta-cell function [28].

Both RES and melatonin (MEL) will activate SIRT1 expression. Endogenously produced melatonin decreases with aging and is responsible for some of the deleterious effects associated with aging. MEL and RES are both found in some foods and may have anti-aging effects. CR will also activate sirtuin expression [29].

### Resveratrol and memory loss

RES has been shown to inhibit memory loss and mood dysfunction which can occur during aging. This occurs via increased hippocampal neurogenesis and microvasculature and inhibition of glial activation in small rats. RES supplementation resulted in improved learning in the rats. This has been associated with increased angiogenesis and decreased astrocytic hypertrophy and decreased microglial activation in the hippocampus. The beneficial effects of RES may be due to effects on the hippocampus plasticity and suppression of chronic low-level inflammation. This study suggests that addition of RES to the diets of adults could have promise in preventing some of the deleterious effects that aging has on memory and other related functions [30].

Importantly, the effects of RES have been examined on human subjects. RES was shown to enhance memory performance and glucose metabolism in older heathy adults. 23 patients (50-75 years old) consumed RES, while 23 received placebo for 26 weeks. Patients treated with RES also exhibited decreased glycated hemoglobin (HbA1c) and body fat, and increases in leptin in comparison with control patients treated with placebo [31].

### Resveratrol and Alzheimer’s disease-enhancing cerebral circulating function

RES may have neuroprotective roles in AD and may improve memory function in dementia. In studies with rats in a water maze model, RES inhibited the expression of inflammatory cytokines (TNF-alpha and IL-1beta) in the older rats. The studies point to potential benefits of RES in terms of memory decline which occurs during aging [32]. In a rat model of ibotenic acid induced AD, RES was shown to ameliorate memory deficiency associated symptoms, alleviating cholinergic pathways, and reduce oxidative stress [33]. In a mouse model with beta amyloid (Abeta1)-42-induced cognitive impairment, RES was shown to inhibit phosphodiesterase-4 related signaling which is important in beta amyloid-induced memory impairment [34]. miR-603 has been associated with AD risk and pathogenesis. miR-603 binds the 3′untranslated region of low density lipoprotein-related protein-associated protein 1 (LRPAP1) mRNA and downregulates the mRNA and protein [35]. LRPAP1 acts as a chaperone to aid in the trafficking of the LDL receptor family members, LRP1 and LRP2. LRPAP1 and LRP1 have opposite effects. miR-603 decreases LRPAP1 levels while increasing LRP1 levels. miR-603 and the associated rs11014002 single nucleotide polymorphism (SNP) may serve as a protective factor against AD. The SNP promotes the biogenesis of mature miR-603 and has a protective effect with regards to AD risk. The expression of the LRPAP gene is down regulated by miR0603 binding the 3′UTR. In contrast, miR-603 increases LRP1 expression. LRP1 and LRPAP1 are involved in Abeta protein clearance and AD pathogenesis.

### Resveratrol and aging in the eyes

The roles of RES and SIRT1 in controlling the resistance to oxidative stress in lens epithelial cells (LECs) have been examined. RES is an activator of SIRT1. Cells were treated with RES and an inhibitor of SIRT1, nicotinamide, and incubated with H_2_O_2_ which induces oxidative stress. The effects of this treatment on SIRT1, TP53 and acetylated TP53 were examined. Upon H_2_O_2_ treatment, SIRT1 was increased and increased further upon RES treatment. RES prevented the morphological changes and apoptosis induced by H_2_O_2_ treatment and promoted proliferation and a decrease in acetylated TP53. Nicotinamide treatment, on the other hand, enhanced apoptosis under oxidative stress conditions, decreased proliferation and resulted in increased acetylated TP53. The TP53 inhibitor pifithrin-alpha (PFT-alpha) prevented the effects of nicotinamide [36].

The ability of SIRT1 to serve as a regulator of aging in the retina has been examined after 1 month and 19 months after oral administration of RES to rats. The expression of SIRT1, brain derived neurotropic factor (BDNF) and tropomyosin receptor kinase B (TRKB) was examined in these studies. SIRT1 was determined to decrease in aged retinas in comparison to young retinas. The expression of BDNF, TRKB and SIRT1 and b-wave amplitude was increased in the retinas upon RES treatment. More apoptosis was detected in the aged rat retinas which did not receive RES than in the retinas of the rats which did receive RES. These studies suggest that RES treatment increased SIRT1 levels may prevent some of the deleterious effects associated with retina aging [37]. The effects of RES on macular degeneration were examined. RES had effects on TGF-beta and hypoxia-induced VEGF secretion by human retinal pigment epithelial cells (HRPE) that were derived from elderly patient’s eyes. Treatment with cytokines such as: interferon-gamma (IFN-gamma), TNF-alpha and IL-1beta resulted in increased expression of vascular endothelial growth factor-A (VEGF-A) and VEGF-C in the eye cultures. RES suppressed the secretion of the VEGF-A and VEGF-C in the eye cultures. RES was also demonstrated to suppress the mobility of the eye cells in scratch wound closure assays. Overexpression of VEGF is often associated with macular degeneration and one the treatments is by ocular injection of a monoclonal antibody such as bevacizumab which is directed to VEGF. RES may be beneficial in the treatment of macular degeneration and diabetic retinopathy [38].

miR-320a has been shown to target the VEGF signaling pathway. Elevated miR-320a expression was determined to reduce cardiac microvessel density. This resulted in decreased cardiac function in mice treated with doxorubicin. VEGF-A is a target of miR-320a. These results indicate that miR-320a and VEGF-A play key roles in cardiotoxicity induced by doxorubicin. Targeting miR-320a may alienate some of the cardiotoxicity associated with doxorubicin treatment by suppressing miR-320c [39].

### Resveratrol and hearing loss

The expression of TP53/miR-34a/SIRT-1 axis was examined during aging in cochlear hair cells which are involved in hearing and deafness. Increased levels of miR-34a, acetylated TP53 and apoptosis were observed in cochlear hair cells from aged C57BL/6 mice while the levels of SIRT-1 decreased. Overexpression of miR-34a was determined to inhibit SIRT1 expression in the inner ear HEI-OC1 cell line. This resulted in increased TP53 acetylation and apoptosis. In contrast, suppression of miR-34a increased SIRT1 expression and decreased TP53 acetylation and apoptosis. RES was shown to rescue the detrimental effects of miR-34a overexpression on HEI-OC1 cells and had positive effects with regards to protection from hearing loss and hair cell loss in C57BL6/J mice [40].

### Potential of resveratrol in cancer and CSCs

**Figure 1:**
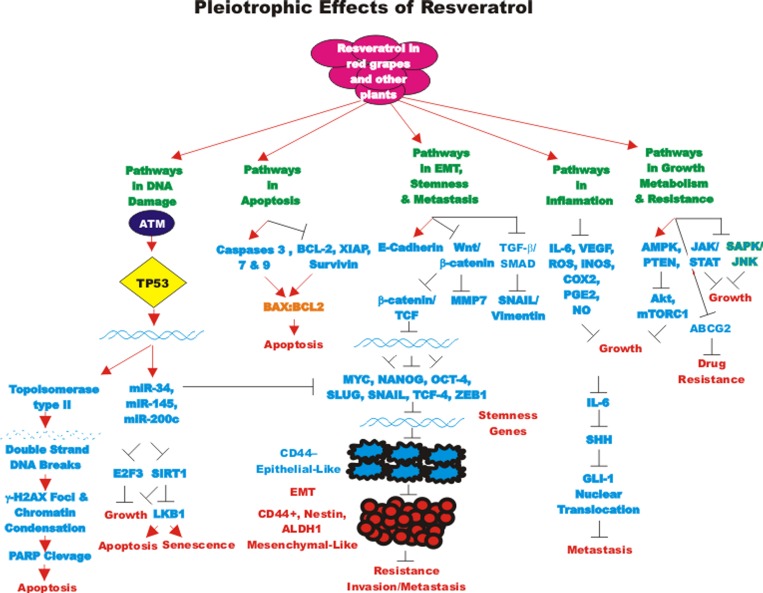
Pleiotropic effects of resveratrol on signaling pathways involved in cell growth Some of the pathways affected by RES are indicated. This diagram focusses on signaling pathways predominately involved in aging and cancer. Red arrows indicate induction of an event; black closed arrows indicate suppression of an event.

The abilities of RES to inhibit various types of cancer have been examined. RES has been shown to stimulate the differentiation of CSCs as well as induce apoptosis and autophagy. In some cases, RES has been shown to synergize with chemotherapeutic drugs and inhibit proliferation. We will briefly summarize some of the different types of cancer that RES has been examined on. We will focus primarily on RES and CSCs. An overview of the pleiotropic effects of RES on cancer pathways is presented in Figure 1.

### Effects of resveratrol on brain cancer

The effects of RES on the induction of autophagy and apoptosis were examined in glioblastoma (GBM) CSCs. RES induced autophagosome formation in three GBM cell lines. The expression of proteins associated with autophagy such as: autophagy protein 5 (Atg5), beclin-1 and Microtubule-associated proteins 1A/1B light chain 3B (LC3-II) were increased after RES treatment. Suppression of the autophagy induced by RES resulted in apoptosis. Suppression of both autophagy and apoptosis was required to block the toxicity of RES. RES suppressed the sphere-forming capacity of the GMB cells and the percentage of CD133 and octamer-binding transcription factor-4 (OCT4) positive cells [41].

Increased levels of signal transducer and activator of transcription 3 (STAT3) have been detected in CD133+, GMB CSCs. Suppression of STAT3 by either pharmacological or genetic approaches inhibited the CSC properties of GMB CD133+ cells. RES was determined to inhibit STAT3, induce apoptosis, suppress the stemness gene signature and induced differentiation. RES or sh-STAT3 treatment was determined to suppress GBM CSC survival in mice and their combined treatment synergistically increased radiosensitivity [42].

The effects of RES on human U87MG cells and primary human glioblastoma cultures were examined. RES suppressed the invasive growth and stimulated differentiation. RES decreased NESTIN and stimulated glial acidic fibrillary protein and betaIII-tubulin and senescence [43].

RES inhibited the self-renewal and tumor-initiating capacity of patient-derived glioblastoma stem cells (GSC). RES was determined to suppress the expression of NANOG through proteasomal degradation which could be inhibited by the proteasome inhibitor MG132. TP53 activation is important in the suppression of NANOG after RES treatment. RES treatment activated TP53 and p21^Cip1^. Suppression of NANOG inhibited self-renewal and tumor-forming capacity of the GSCs. These studies indicated that RES could induce GSC differentiation and NANOG was important in stemness [44].

The effects of RES have been compared on GSCs and normal neural stem cells (NSCs). RES was determined to block GSC proliferation, while it did not appear to have any effects on NSCs. SIRT1 and SIRT3 were expressed in both GSCs and NSCs, however, SIRT2 was only expressed in GSCs. Inhibition of SIRT2 suppressed the anti-proliferative effects of RES [45].

The effects of combining RES with radiation have been examined in the radioresistant GBM SU-2 cell line. RES was demonstrated to inhibit proliferation, enhance radiosensitivity, suppress neural stem cell markers and induce differentiation in SU-2 cells. The combination of RES and radiation resulted in increased autophagy, apoptosis in *in vitro* and *in vivo* models. In addition, RES treatment suppressed repair of radiation-induced DNA damage [46].

### Effects of resveratrol on breast cancer

RES inhibited the proliferation of CSCs isolated from MCF-7 and SUM159 breast cancer cells. RES also reduced the presence of mammospheres in these two cell lines and inhibited tumor formation of the mammospheres in NOD/SCID mice. RES also induced autophagy in the breast mammospheres as determined by analysis of GFP-LC3-II puncta formation assay and the levels of LC3-II, Beclin1 and Atg 7 proteins. RES suppressed WNT/beta-catenin pathway expression [47].

The abilities of RES and the chemically-related pterostilbene to combine and restore the ERalpha expression in ERalpha-negative breast cancer have been examined. After combined treatment with RES and pterostilbene, increased levels of active acetyl-H3, acetyl-H3lysine9 (H3K9) and acetyl-H4 binding to the ERalpha promoter analysis were observed by chromatin immunoprecipitation analysis (ChIP). The combined treatment also resulted in changes in HDAC and histone acetyl transferase activity (HAT) as reduction in DNMT enzyme activity and 5-methylcytosine levels were observed in the normally ER- MDA-MB-157 breast cancer cells. The combined treatment also rendered the cells sensitive to the ER antagonist tamoxifen. This combination approach may be a safer alternative to restore ER expression and sensitivity to anti-ER based therapeutics in certain breast cancer patients [48].

The effects of the RES analog 4,4′-dihydroxy-trans-stilbene (DHS) were examined on normal mouse and human fibroblasts and human breast cancer cells. DHS was determined to inhibit the growth of human fibroblast cells more than normal RES. DHS was determined to suppress chemically-induced (3-methylcholanthrene plus 12-O-tetradecanoylphorbol-13-acetate) transformation of BALB/c-3T3 and invasion of MCF-7 breast cancer cells. Interestingly, DHS was determined to inhibit both anchorage-dependent and anchorage-independent growth of MCF-7 growth more efficiently than RES. DHS resulted in pRb inhibition and increased expression of TP53 and p21^Cip-1^. DHS was determined to reduce matrix metalloproteinase-2 (MMP2) and MMP9 expression, decreased adhesion to the extracellular matrix, migration and invasion [49].

Dihydrotestosterone (DHT) can stimulate the growth of breast cancer cells. DHT can interact with both ERalpha and integrin alphavbeta3. RES can induce TP53-dependent apoptosis via integrin alphavbeta3. ERK1/2 can transduce, in part, the signals mediated by RES and DHT, while DHT promotes proliferation, RES induces apoptosis. The effects of RES and DHT were compared on MCF-7 (ERalpha+) and MDA-MB-231 (ERalpha-) breast cancer cells. DHT was determined to inhibit phosphorylation of TP53 at S15. DHT also inhibited the nuclear TP53/COX-2 complex and TP53 transcriptional program. Thus, the receptor sites necessary for the effects of DHT and RES are discrete. While they both result in activation of ERK1/2 they have different effects. These studies also indicate the importance of the presence and absence of DHT in studies evaluating the effects of RES [50].

The effects of RES were examined in paclitaxel-resistant MDA-MB-231 cells. RES was determined to inhibit proliferation and stimulate apoptosis and cellular senescence in both paclitaxel-resistant and paclitaxel-sensitive MDA-MB-231 cells indicating that the effects of RES were independent of the paclitaxel-sensitivity status of the MDA-MB-231 cells. The resistant cells expressed increased levels of multidrug resistance-1 (MDR1) and cytochrome P4502C8 (CYP2C8) genes [51].

### Effects of resveratrol on colorectal cancer (CRC)

The diet is very important in the development of cancer, especially CRC. CRC CSCs are resistant to conventional chemotherapeutic drugs. The Western diet is becoming more and more associated with obesity which results in elevated levels of insulin and IGF-1. This may contribute to activation of the PI3K/PTEN/Akt/mTORC1 and WNT/beta-catenin pathways which contributes to CRC CSCs as well as premature aging. These CRC CSCs are often resistant to small molecule inhibitors which target these signaling pathways. A current focus of nutrition research is the effects of dietary bioactive compounds such as: CUR, grape seed extract, lycopene (present in tomatoes and other red vegetables), RES and others [52].

SIRT1 is an important subcellular target for RES. Depending on the cell system and conditions, RES can act as a tumor promoter or a tumor suppressor. The effects of RES on the SIRT1 were examined in CRC cells. RES suppressed proliferation in two CRC cell lines, this was accompanied with a decrease in Ki-67 expression which was dependent upon SIRT1 activity. RES downregulated nuclear localization and activity of NF-kappa-B which resulted in decreased expression of MMP9 and C-X-C chemokine receptor type 4 (CXCR4), two proteins associated with metastasis. SIRT1 was determined to interact with NF-kappaB. RES can effect SIRT1 expression which can regulate negatively NF-kappaB activity [53].

RES has been determined to suppress proliferation, migration, invasion and induce apoptosis in CRC cell lines, such as HT-29 (TP53+) and HCT-116 (TP53-) CRC cell lines.

The expression of miR-34c is increased by RES in CRC and this expression was in part responsible for the effects of RES. RES also sensitized the CRC cells to oxaliplatin in a miR-34a-dependent fashion. In xenograft studies, treatment with either RES or oxaliplatin suppressed tumor growth and their combined treatment was synergistic. In this xenograft system, RES increased miR-34 levels and also reduced the level of IL-6 which normally promotes CRC progression. The effects of RES were increased in the presence of functional TP53, indicating that RES and TP53 synergized [54].

The pharmacological properties of RES can be enhanced by nanoencapsulation. Normally the solubility and stability of RES is poor. The effects of nanoencapsulation of RES have been examined in CRC and other model systems and have shown enhanced delivery [55].

A common problem with 5-flurouracil (5FU) treatment of CRC patients is chemo resistance. The effects of RES on 5FU-sensitive (HCT116, SW480) and their corresponding isogenic 5FU-chemoresistant derived CRC cell clones (HCT116R, SW480R) have been examined. Interestingly, RES blocked proliferation of all four cell lines and synergized with the effects of 5FU. RES was determined to suppress many gene products associated with EMT such as decreased vimentin and SLUG expression but increased E-cadherin expression. RES down-regulated NF-kappaB activation and nuclear translocation resulting in inhibition of NF-kappaB regulated gene expression which included MMP-9 and caspase-3. This occurred by inhibition of IkappaBalpha kinase which controls NF-kappaB activity [56].

The abilities of RES to suppress the cisplatin-resistance of CRC cells have been examined. The effects of addition of 15 micrograms/ml RES were determined on CRC cells treated with 5 and 20 micrograms/ml cisplatin. RES could induce anti-proliferative and pro-apoptotic effects in both the drug-sensitive and cisplatin-resistant HCT-116 cells. The cellular uptake of cisplatin was improved in the presence of RES [57].

The effects of RES also have been examined in combination with oxaliplatin in the HCT116 CRC cell line. The effects of RES and oxaliplatin were examined on the inhibitor of apoptosis protein survivin which is a key anti-apoptotic protein. While oxaliplatin treatment decreased the levels of survivin, the combined treatment of oxaliplatin and RES restored survivin, BCL-2 and caspase levels. In these studies, the apoptotic-inducing effects of oxaliplatin were decreased when RES was added to the HCT116 cell line. Thus, in the HCT116 cell line, RES had an anti-chemosensitizing effect when added with oxaliplatin. These and other studies demonstrate the caution that needs to be applied to studies with using RES on CRC and other cancer types [58].

The abilities of RES to enhance the anti-proliferative effects of etoposide in HepG2 liver cancer cells and CRC HCT-116 cells were determined. Both cell lines have functional TP53. RES inhibited proliferation in both cell lines. The combination of RES and etoposide resulted in greater anti-proliferative effects in HCT-116 than just after etoposide treatment by itself whereas this effect was not observed in HepG2 cells. TP53 was detected at higher levels after RES and etoposide treatment. Pre-incubation of both cell lines with RES, increased the level of etoposide-induced TP53. Thus, RES appeared to promote the effects on TP53 [59].

RES can induce AMPK which results in inhibition of the drug transporter MDR1 in oxaliplatin-resistant (L-OHP) HCT116/L-OHP CRCs. This prevents NF-kappaB activation and levels and suppresses cAMP-responsive element transcriptional activity. AMPKalpha siRNA transfection could reverse the effects of RES on MDR1 expression and cAMP-responsive element-binding protein (CREB) phosphorylation [60].

RES induces chromatin condensation and TP53 and the cleavage of poly [ADP-ribose] polymerase 1 (PARP-1) in CRC cells. The *TP53* gene status was determined to affect the sensitivity to RES as CRC cells with WT TP53 (HCT-116/p53 WT) were more sensitive to RES than cells with mutant *TP53* (HCT-116/p53−/−). RES induced double strand DNA breaks by interfering with type II topoisomerase. This was ascertained by determining the extent of gamma-H2AX foci present after treatment with RES. The DNA damage was determined to be due to type II topoisomerase poisoning. Treatment of HCT-116 cells with RES was determined to result in activation of Ataxia Telangiectasia Mutated (ATM) kinase which in turn activated TP53 [61].

RES suppresses EMT via the TGF-beta/SMAD pathway in CRC LoVo cells *in vitro* and in animal experiments *in vivo*. The TGF-beta/SMAD pathway regulated SNAIL/E-cadherin expression. RES inhibited CRC metastasis into the lung in colon cancer metastatic tumor model after tail vein injection as well the development of lung and liver tumors after orthotopic injections. *In vitro* studies showed that TGF-beta promoted EMT and invasion and reduced E-cadherin but elevated vimentin expression. RES inhibited cancer cell invasion and induced E-cadherin and repressed EMT, vimentin, the TGF-beta/SMAD pathway and SNAIL [62].

The effects of RES and the DNA cross-linker mitomycin C have been examined in CRC primary CRC cells isolated from resected tumors. The combination of RES and mitomycin C suppressed proliferation better than either drug by itself. This treatment resulted in a significant up-regulation of p21^Cip-1^ expression [63].

The effect of RES and 5-aminosalicylic acid (Mesalazine) on JAK/STAT activation have been examined in the HT-29 CRC line. Mesalazine is an anti-inflammatory drug used to treat patients with bowel disorders, including inflammatory bowel disease. RES reduced nitric oxide (NO) and prostaglandin E2 (PGE2) production, inducible nitric oxide synthase (iNOS) and cyclooxygenase-2 (COX-2) expression and ROS production which were induced by treatment of HT-29 cells with the inflammatory cytokines (IL-1alpha, TNF-alpha, IFN-gamma). RES was determined to decrease activation of the JAK/STAT pathway but did not result in degradation of I-kappaB-alpha. RES also inhibited stress-activated protein kinase (SAPK)/c-Jun N-terminal kinase (JNK) pathway activation [64].

In a different study, RES was shown to inhibit the proliferation of human HCT116 CRC cells *in vitro* as well as in tumor xenograft studies *in vivo*. RES was determined to upregulate phosphatase and tensin homolog (PTEN) expression and decrease the expression of activated Akt. In HCT116 cells, PTEN inhibits Akt signaling and proliferation. Moreover, decreased levels of beta-catenin were also detected after RES treatment [65].

RES was shown to decrease WNT/beta-catenin pathway activity and the downstream targets c-Myc and MMP-7 in CRC cells. RES also decreased the expression of long non-coding metastasis associated lung adenocarcinoma transcript 1 (RNA-MALAT1) in the LoVo and HCT116 CRC cells. This resulted in decreased nuclear localization of beta-catenin and suppressed WNT/beta-catenin signaling. These events were linked with suppression of CRC invasion and metastasis [66].

Treatment of CRC cells with RES resulted in decreased expression of transcription factor 4 (TCF4), which is a critical effector molecule of the WNT/beta-catenin pathway. The half-life of the TCF4 protein was decreased after treatment with RES. In contrast, RES did not affect the transcription rate of the TCF4 gene. RES increased the S/T phosphorylation of TCF4. These phosphorylation events were determined to be due to ERK1/2 and p38^MAPK^ [67].

RES can induce anti-proliferative effects on CRC cells via the miR-34a/E2F3/Sirt1 pathway. The effects of RES, epigallocatechin-3-gallate (EGCG), and alpha-mangostin (alpha-M) were examined on three CRC lines in the presence and absence of 5-FU. These natural products were determined to suppress the PI3K/PTEN/Akt/mTORC1 pathway. Alpha-M was determined to be the most potent PI3K/PTEN/Akt/mTORC1 pathway inhibitor and suppressed Raf/MEK/ERK pathway. Combined treatment of RES and 5-FU resulted in a synergistic inhibition of cell growth and induction of apoptosis and the Raf/MEK/ERK pathway was suppressed in DLD-1 CRC cells. RES increased the expression of miR-34 which resulted in the decreased expression of E2F3 and SIRT1 [68].

### Effects of resveratrol on head and neck cancer

The effects of RES on the CSC properties of head and neck cancer-derived tumor-initiating cells (HNC-TICs) were examined. RES was determined to downregulate ALDH1 and CD44 in HNC-TICs in a dose-dependent fashion. RES also inhibited OCT4, NANOG and NESTIN expression in the sphere forming HNC cells. In mouse experiments, RES was shown to inhibit tumor growth, stemness and EMT markers. RES synergized with chemotherapy in suppression of the growth of HNC-TICs [69].

### Effects of resveratrol on leukemia

RES suppressed phosphorylated liver kinase B1 (pLKB1) and induced senescence and apoptosis in acute myeloid leukemia (AML) KG1a cells. This occurred via SIRT1 which is a regulator of LKB1 (aka serine/threonine kinase 11 [STK11]) which resulted in senescence and apoptosis [70].

RES has been determined to decrease IL-6-induced Sonic hedgehog homolog (SHH) signaling in AML. The plasma levels of IL-6 and IL-1beta were higher and lower respectively in AML patients than in normal donors. The expression of SHH was determined to be higher in bone marrow and peripheral blood mononuclear cells prepared from AML patients. IL-6 was determined to increase SHH and GLI-1 expression in HL-60 cells. IL-6 also increased cell viability. RES decreased SHH expression, GLI-1 nuclear translocation and cell viability in IL6-treated HL-60 cells. RES was determined to synergize with the SHH inhibitor cyclopamine in inhibiting growth [71].

### Effects of resveratrol on lung cancer

RES has been shown to inhibit the secretion of IL-6 and VEGF from A549 lung cancer cells when they were co-cultured with adipose-derived mesenchymal stem cells [72].

The ability of combined RES and metformin (MET) treatment to protect A549 lung cancer cells from ultraviolet C (UVC)-induced damage was determined. MET was determined to inhibit the UVC-mediated increase in TP53 expression and suppressed expression of gamma-H2AX and phosphorylated check kinase-2 (P-Chk2). MET also induced DNA repair and cell cycle arrest and decreased levels of cyclin E/cyclin-dependent kinase 2(cdk2)/RB and cyclin B1/CDK1. Treatment with RES, by itself, was less effective in suppressing UVC-induced responses. Combined RES and MET treatment resulted in a synergistic response in terms of decreased TP53, gammaH2AX and P-Chk2 expression. Thus, the combination of RES and MET might suppress some of the aging effects elicited by UVC-induced DNA damage [73].

RES has been shown to regulate the expression of lncRNAs in lung cancer. 21 lncRNAs were shown to have increased expression after RES treatment and 19 lncRNAs were detected at lower levels in A549 lung cancer cells. The AK001796 lncRNA was determined to be overexpressed in lung cancer cell lines and tissues but it was detected at lower levels in RES-treated cells. The AK001796 lncRNA functioned as an oncogene. Suppression of AK001796 lncRNA inhibited cell growth and promoted cell cycle arrest [74].

### Effects of resveratrol on hepatocellular carcinoma

The effects of RES on hexokinase 2 (HK2) were examined in HCC. HK2 can change the metabolic properties of cancer cells to promote growth in conditions of low oxygen concentrations. This can result in aerobic glycolysis. Aerobic glycolysis was observed in four HCC lines as opposed to normal hepatic cells. Treatment with RES was observed to sensitize the HCC cell lines to apoptosis and these effects were reversed upon treatment with glycolytic inhibitors. RES treatment resulted in a decrease in HK2 and increased mitochondrial-induced apoptosis. RES could enhance treatment with sorafenib resulting in growth inhibition and promoting apoptosis in HCC xenografts in mice [75].

The effects of tanshinone IIA (Tan IIA) have been examined in combination with RES on the induction of cytotoxicity, cell-cycle arrest, apoptosis, and DNA fragmentation in HepG2 HCC cells. Tan IIA and RES were determined to synergize in inducing apoptosis. The levels of apoptosis induced with Tan IIA and RES were similar to the amounts induced by cisplatin [76].

RES has been shown to induce apoptosis in HepG2 cells by induction of caspase-3 and -9, TP53 and increasing the BAX/BCL2 ratio. The ability of matrine, a natural component extracted from the traditional Chinese medical herb *Sophora flavescens Ait* to synergize with RES was determined also. Matrine could synergize with RES in inhibiting cell growth and promoting apoptosis. This synergy in the induction of apoptosis was attributed to the induction of caspases -3 and -9, downregulation of survivin, induction of ROS and alteration of the mitochondrial membrane potential [77]. The effects of RES on VEGF expression and proliferation of HCC cells were determined. RES inhibited VEGF expression in the HepG2 cells [78].

RES was determined to sensitize HepG2 cells to TNF-related apoptosis-inducing ligand (TRAIL)-induced apoptosis. RES induced an increase in phospho-AMPK levels and a decrease in survivin levels. RES was determined to increase TRAIL sensitivity by decreasing survivin expression [79].

The effect of RES on the induction of apoptosis in SMMC-7721 cells were determined. RES could activate caspases-3 and -9 and JNK whereas it downregulated activated ERK. RES also was effective in xenograft models with SMMC-7721 cells [80].

RES has been shown to suppress the STAT3 signaling pathway in HepG2 cells. This resulted in the suppression of proliferation when the cells were cultured in medium containing high glucose. This was determined to be mediated in part by SIRT1 expression. When HepG2 cells were cultured in medium containing 25 millimolar glucose, exposure of the cells to 100 micromolar Res suppressed proliferation and STAT3 signaling [81].

Res has also an anti-metastatic effects in HCC through inhibition of phosphorylation of the JNK 1/2 pathway and SP-1 DNA binding activities, causing a downregulation of urinary-type plasminogen activator (u-PA) expression [82].

RES prevented diethylnitrosamine (DEN)–induced liver tumorigenesis in rats by suppressing oxidative stress and inflammatory response mediated in part by hepatic nuclear factor E2–related factor 2 (Nrf2) [83]. RES in combination with CUR has synergistic antiproliferative effects in HCC Hepa1-6 cells [84].

### Effects of resveratrol on nasopharyngeal cancer

The effects of RES on nasopharyngeal carcinoma (NPC) CSCs were examined. Interestingly the NPC CSCs were determined to have undergone a metabolic shift as they relied on glycolysis for energy. RES was determined to shut off the metabolic shift and increase ROS levels and depolarized mitochondrial membranes. These biochemical events were associated with a reversion of the CSC-associated properties of the cells. RES also was found to inhibit the CSC properties of the cells by activating TP53 and inducing miR-145 and miR-200c expression, which are normally downregulated in NPC CSCs [85].

### Effects of resveratrol on ovarian cancer

The effects of RES on the induction of ROS has been examined in A2780 ovarian CSCs. RES was determined to kill A2780 ovarian CSCs in a fashion independent of ROS. However, ROS inhibited the self-renewal capacity of A2780 ovarian CSCs which could survive the RES treatment [86].

### Effects of resveratrol on pancreatic cancer

RES has also been suggested to have anti-cancer effects on pancreatic cancer cells. RES inhibited proliferation, invasion and metastasis and induced apoptosis in pancreatic cancer cells and suppressed proliferation and viability of pancreatic CSCs. RES also could chemosensitize pancreatic cancer cells and have effects on diabetes [87].

Human pancreatic cells which are CD133+, CD44+, CD24+, ESA+ and ALDH+ are enriched in pancreatic CSCs. They also express higher levels of NANOG, OCT-4, NOTCH1, MDR1 and ATP Binding Cassette Subfamily G Member 2 (ABCG2) than primary pancreatic cells and normal pancreatic tissues. CSCs were derived from *Kras(G12D)* transgenic mice. These CSCs also express higher levels of NANOG and OCT-4 than the cells from control mice. RES was determined to suppress growth and development of pancreatic cancer in the *Kras(G12D)* mice. RES activated caspases -3, -7 and apoptosis by inhibiting BCL-2 and XIAP in the pancreatic CSCs. RES also inhibited: NANOG, SOX-2, c-MYC, OCT-4 and ABCG2 in the pancreatic CSCs. Genetic suppression of NANOG by shRNA was determined to enhance the effects of RES on pancreatic CSCs self-renewal. RES also inhibited pancreatic CSC migration, invasion and the expression of genes associated with EMT including: ZEB-1, SLUG and SNAIL [88].

### Resveratrol and thyroid cancer

Combined RES and valproic acid treatment has been shown to inhibit the growth, stem cell marker expression, aldefluor expression and invasiveness of two spheroid thyroid carcinoma CSC lines derived from anaplastic thyroid cancers. Although valproic acid is predominately used in the treatment of epilepsy and bipolar disorder and to prevent migraine headaches, it is also being examined for its effects on cancer and is in at least 83 clinical trials with various cancer patients. Valproic acid may inhibit histone deacetylase and this is one of the reasons this drug is being examined in cancer settings. RES and valproic acid treatment resulted in an increase in thyroid differentiation marker expression and apoptosis [89].

### Summary of resveratrol

The anti-aging, anti-cancer, anti-neurological effects of RES have been examined in many different diseases and environments. While there have been many beneficial effects postulated to be induced by consumption of RES, the concentrations required to obtain those health benefits may be high. Although, there probably are many beneficial health effects of consumption of products rich in RES.

### Overview of effects of curcumin

CUR has also been reported to have many beneficial health properties ranging from anti-aging, anti-cancer, anti-hypertensive, anti-inflammatory and anti-neurological effects. The world-wide market for CUR is predicted to be close to $100 million by 2022 (http://www.grandviewresearch.com/industry-analysis/turmeric-extract-curcumin-market). CUR is commonly obtained as an extract from *Curcuma longa* (Turmeric). There are other compounds present in the extract from *Curcuma longa* which are related to CUR, they are called Curcuminoids. 60-70% of the turmeric extract consists of CUR, 20-27% of the turmeric extract consists of demethoxy curcumin and 10-15% of the turmeric extract consists of bisdemethoxycurcumin [90]. Together these components make up 1-6% of turmeric by weight.

### Curcumin and aging

CUR may have anti-aging properties. The effects of CUR may be mediated, in part, by their effects on sirtuins. High doses of CUR (2.5-10 micromolar) have been shown to induce the senescence of cancer cells and cells involved in building the vasculature. The effects of lower doses of CUR (0.1 and 1 micromolar) on vascular smooth muscle (VSMC) and endothelial (EC) cells have been examined. VSMCs were not protected from replicative senescence or premature senescence induced by doxorubicin with low doses of CUR. CUR treatment did have some effects on increasing the levels of sirtuins. Thus, low doses of CUR could increase sirtuins levels in the absence of delaying the senescence of VSMCs [91].

The ability of CUR to alter the expression of the mitochondrial uncoupling protein 2 (UCP2) in old and young rats (UCP2−/− and WT) was determined as this protein plays critical roles in regulating ROS production. These experiments were performed to determine whether dietary CUR has positive effects on aging-related cerebrovascular dysfunction by increasing UCP2 expression. CUR was determined to reduce ROS production in UCP2 WT but not in UCP2−/− aging mice. CUR restored the cerebrovascular endothelium-dependent vasorelaxation that was impaired in the older rats. Thus, these studies demonstrated that CUR could improve cerebrovascular dysfunction which occurs during aging [92].

The effects of CUR and MEL on age-related carbonyl content of liver in young and old mice were determined. Carbonyl content in the liver is detected at increased levels during aging. CUR and MEL treatment decreased the carbonyl content found in the livers of both the young and old mice [93].

### Curcumin and lifespan

In *Drosophila* models, CUR has been shown to prevent the accelerated aging that is normally observed after irradiation by reduction of oxidative stress [94]. CUR was also shown by a different group to enhance the *Drosophila* reproductive lifespan as well as prolong the viability of the offspring [95].

Tetrahydrocurcumin (THC) has been shown to increase the life span of certain organisms including: nematodes, *Drosophila* and mice. Interestingly in the nematode model, CUR reduced the production of ROS. The following genes: odd-skipped-related 1 (*osr-1* a transcription factor), dual specificity mitogen-activated protein kinase kinase 4 (MAP2K4, *sek-1,* a serine/threonine (S/T)kinase), dual specificity mitogen-activated protein kinase kinase 1 (*mek-1*), skinhead-1 (*skn-1* encodes a beta-glucan synthesis-associated protein), Ca2+/calmodulin-dependent protein kinase II (CaMKII, *unc-43*, encodes a S/T kinase, Sirtuin2 (silent mating type information regulation 2) 2 (*sir-2.1*), and phosphatidylinositol 3-kinase age-1 (*age-1*) were demonstrated to be responsible for the effects of CUR on lifespan in nematodes. In *Drosophila*, superoxide dismutase activity was required for increased lifespan. In addition, decreased lipofuscin and malondialdehyde levels were required for increased lifespan in *Drosophila*. CUR also upregulated the expression of superoxide dismutase (*SOD*) genes as well as decreased the expression of various age-related genes including: *Drosophila* insulin receptor (*dInR*), Attacin-D (*ATTD*, a protein in *Drosophila* that induced upon bacterial infection), defensin (*Def*, a protein induced in *Drosophila* in response to fungal infection), Cecropin B (*CecB*, a protein involved in immune response of *Drosophila*) and bactericidal protein diptericin B (*DptB*, a protein involved in survival response of *Drosophila*) [96].

### Curcumin and senescence

**Figure 2:**
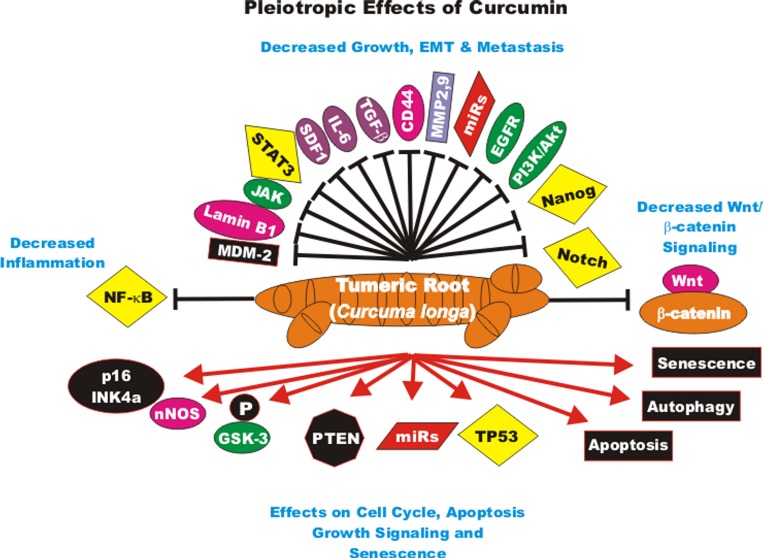
Pleiotropic effects of curcumin on signaling pathways involved in cell growth Curcumin can induce many pathways which may result in suppression of cell growth, induction of apoptosis, autophagy, senescence or inhibition of cell cycle progression. These events are indicated by red arrows. In addition, CUR treatment can result in the suppression of many important proteins which result in decreased growth, EMT, metastasis or inflammation. These events are indicated by black closed arrows.

The effects of CUR on gene expression in cancer-associated fibroblasts obtained from breast cancer patients has been examined. CUR increased the expression of the p16^INK4A^ and other tumor suppressor proteins. In contrast, CUR decreased the activity of the JAK2/STAT3 pathway and many molecules involved in cellular growth and metastasis including: stromal cell-derived factor-1 (SDF-1), IL-6, MMP2, MMP9 and TGF-beta. These effects reduced the levels of alpha-smooth muscle actin (alpha-SMA) which was attributed to decreased migration and invasion of the cells. CUR suppressed Lamin B1 and induced DNA damage-independent senescence in proliferating but not quiescent breast stromal fibroblasts in a p16^INK4A^-dependent manner. The CUR-induced senescence was determined to occur in the absence of an inflammatory secretory phenotype, which was in some cases associated with procarcinogenic properties. Thus, CUR treatment can result in senescence in stromal fibroblasts [97]. An overview of the pleiotropic effects of CUR is presented in Figure 2.

### Curcumin and memory loss diseases

CUR may be useful in the prevention of AD. AD results, in part, from the overproduction and accumulation in the brain of Abeta. CUR has recently been shown to suppress memory decline by suppressing beta-site amyloid precursor protein cleaving enzyme 1 (BACE1= Beta-secretase 1, an important gene in AD) expression which is implicated in beta-amyoid pathology in 5xFAD transgenic mice. CUR was determined to suppress synaptic degradation and improved spatial learning in the 5xFAD mice [98].

The ability of CUR to affect adiposity and obesity-associated cognitive impairment has been examined in mouse models. CUR was found to decrease adiposity and improve cognitive function in a similar fashion as CR in 15-month-old mice. The effects of CUR and CR were positively linked with anti-inflammatory or antioxidant actions [99].

The effects of CUR on the prevention of memory decline have been investigated in old mice. These studies have focused on the effects of CUR on the neuronal nitric oxide synthase (nNOS)/nitric oxide (NO) pathway. Treatment of mice with CUR aided the memory acquisition ability of old mice. CUR treatment increased nNOS expression, acidity and NO concentration [100].

CUR also has been shown to have positive effects on spatial memory by studies with the Morris water maze model with old female rats. In addition, CUR treatment had positive effects on the oxidative stress induced by aging by analysis of malondialdeyde (MDA), protein carbonyl and glutathione levels. CUR treatment resulted in decreased levels of MDA [101].

### Curcumin and macular degeneration

The effects of CUR on age-related macular degeneration (AMD) have been examined in an aging retinal pigment epithelial cell (RPE) model. The effects of CUR on H_2_O_2_-treated RPE cells were determined. CUR treatment improved cell viability and decrease both apoptosis and oxidative stress [102].

Damage induced by oxidative stress to RPE cells may be responsible for the aging of the retina and AMD which can lead to irreversible loss of vision in the elderly. Patient-derived RPEs were isolated by reprogramming T cells from patients with dry-type AMD. The cells were induced to become pluripotent stem cells (iPSCs) and then they were differentiated into RPE cells. Interestingly, the AMD-RPEs had decreased abilities to defend against oxidative stress which may have been responsible for their susceptibility to oxidative damage and AMD. These cells have been used *in vitro* drug screening assays. CUR treatment was determined to cause reduction of ROS in the AMD-RPEs and protected the cells from H_2_O_2_-induced cell death by reduction of ROS levels. CUR also altered the levels of: PDGF, VEGF, insulin like growth factor binding protein 2 (IGFBP-2), heme oxygenase 1 (HO1), superoxide dismutase 2 (SOD2), and glutathione peroxidase 1 (GPX1). Thus, CUR treatment might eventually be useful in the treatment of patients with or susceptible to the development of AMD [103].

### Curcumin and miRs

**Figure 3:**
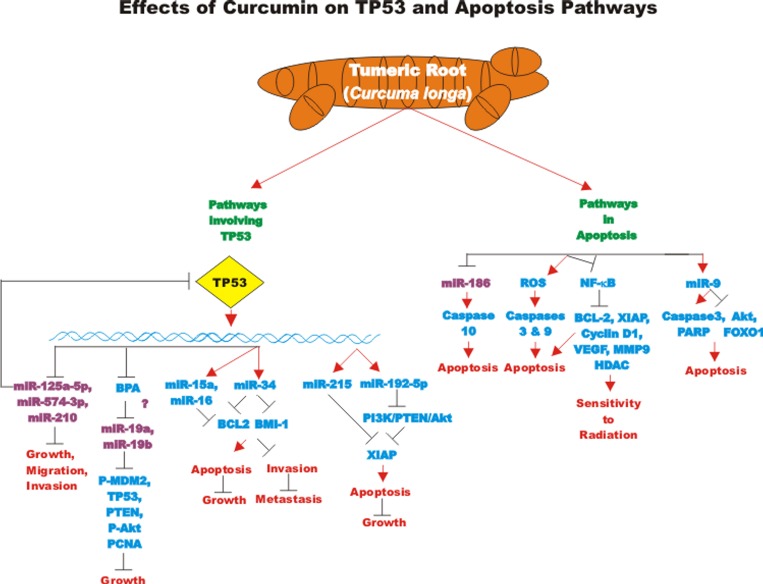
Effects of curcumin on TP53 and apoptosis pathways An overview of the effects of CUR on TP53 and apoptotic pathways and the effects of miRs are indicated. miRs in magenta font indicate oncomirs. miRs in blue font are tumor suppressor miRs. Red arrows indicate induction of an event; black closed arrows indicate suppression of an event.

The effects of many drugs on the expression of various miRs is an important area of scientific investigation as miRs have been shown to play key roles in both positive and negative regulation of gene expression. A diagram of some of the effects of CUR on miRs important in TP53-mediated responses and apoptotic pathways is presented in Figure 3. Certain miRs can act as oncogenes and stimulate abnormal gene expression while other miRs can act as tumor suppressors and inhibit gene expression. Often the promoter regions of miR tumor suppressor genes becomes hypermethylated in cancer cells and the miR is turned off. Approaches to induce the expression of miRs with drugs that induce demethylation are an important area of scientific research.

CUR has been shown to regulate miR-21 in various types of cancer. miR-21 can have effects on PI3K/PTEN/AKT, NF-kappaB, programmed cell death protein 4 (PDCD4) and other signaling pathways. CUR decreases miR-21 levels by increasing miR-21 exosomes. CUR also decreases miR-21 expression by a transcriptional mechanism by binding the promoter region of the miR-21 gene [104].

### Effects of curcumin on bladder cancer

**Figure 4:**
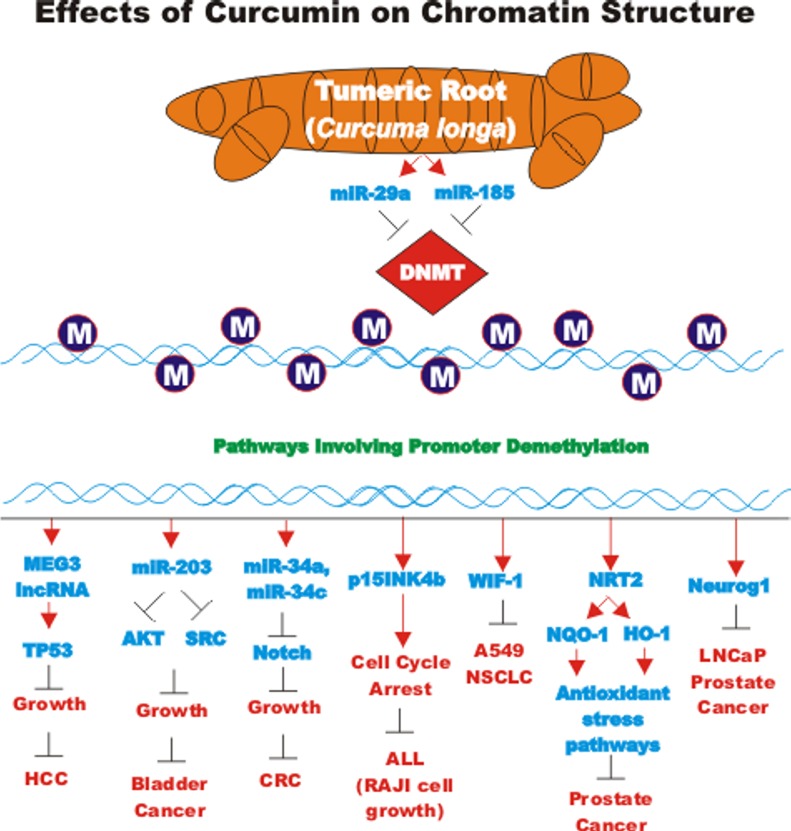
Effects of curcumin on chromatin structure An overview of the effects of CUR on demethylation of genes and the effects on the various genes are indicated. Red arrows indicate induction of an event; black closed arrows indicate suppression of an event. M = methylation of a sequence.

CUR also has been shown to have anti-cancer effects on bladder cancer. CUR has been shown to induce the expression of the tumor suppressive miR-203 in bladder cancer. Normally miR-203 is repressed in bladder cancer perhaps due to hypermethylation of its promoter region. CUR could induce the demethylation of the miR-203 promoter region in bladder cancer cell lines. AKT2 and SRC have been determined to be targets of miR-203 in bladder cancer and their expression was decreased after CUR treatment [105]. In some cancers, CUR has been shown to downregulate the expression of DNA methyl transferase I (DNMT1) and induce DNA hypomethylation [106]. A diagram illustrating the effects of CUR on chromatin structure is presented in Figure 4.

### Effects of curcumin on brain cancers

Demethoxycurcumin (DMC) has been shown to have anti-tumor effects against glioblastoma (GMB). Temozolomide (TMZ) is used for treating GMB, however, TMZ does not prevent GMB recurrence which may be due to the presence of glioma stem cells (GSC). The effects of DMC and TMC on the induction of apoptosis and cell growth of GSC were examined. Combining the two drugs was determined to elicit more anti-GSC effects. Some of the effects were determined alterations in multiple signaling pathways including: JAK/STAT-3 signaling, induction of ROS and caspase-3-mediated apoptosis. Addition of DMC prior to TMZ treatment was determined to be more effective [107].

Interestingly, CUR was shown to both inhibit glioma cell proliferation and induce glioma cells to form spheres which were positive for CD133 and Nestin expression [108]. CUR suppressed G_1_ to S phase transition and enhanced the expression of OCT-4, SOX-2 and SOX-4. The expression of these genes was essential for retaining the stemness of the glioma-initiating cells [108].

**Figure 5:**
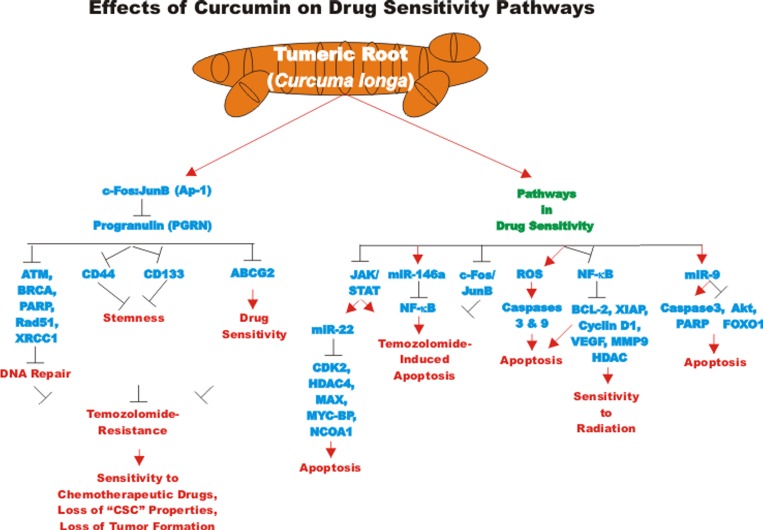
Effects of curcumin on drug sensitivity pathways An overview of the effects of CUR on drug sensitivity pathways and the effects of miRs are indicated. Red arrows indicate induction of an event; black closed arrows indicate suppression of an event.

Progranulin (PGRN) is a protein involved in regulation of cell growth. PGRN is overexpressed in GBM and has been shown to promote temozolomide-resistance by inducing DNA repair and stemness. PGRN upregulated the expression of many genes involved in DNA repair including: Ataxia telangiectasia mutated (*ATM*, a S/T kinase involved in DNA repair response), breast cancer type 1 susceptibility protein (*BRCA1*, a protein involved in DNA repair), PARP, *Rad51* (a protein involved in DNA repair), X-ray repair cross complementing 1 (*XRCC1*) and others. PGRN also upregulated the expression of many genes involved in stemness including: CD44 and CD133 and others involved in drug resistance such as ABCG2. The increased expression of these genes was due to their transcriptional activation by c-FOS/JUNB present in the AP-1 transcription factor complex. CUR can down regulate AP-1 activity. CUR regulates both AP-1 and PGRN activity. Suppression of PGRN inhibited the stemness, temozolomide-resistance and tumor formation of the GBM cells and sensitized the cells to temozolomide. These results point to the importance of the AP-1/PGRN pathway in GBM cells and point to the usefulness of CUR as an adjuvant in GBM therapy [109]. A diagram illustrating some of the effects of CUR on chemoresistance in brain cancer is presented in Figure 5.

CUR may exert anti-tumor effects on glioblastoma CSC by augmenting the apoptotic-inducing effects of ceramide. It has been postulated that CUR might also increase the effects of the chemotherapeutic drug lomustine (an alkylating agent) which is used in second line therapy for glioblastoma [110].

miR-21 can also have effects on migration, invasion and apoptosis of glioma cells. Migration-prone U251-P10 and U87-P10 lines were derived from U251 and U87 parental glioma lines. VEGF and intracellular adhesion molecule-1 (ICAM-1) were detected at higher levels in the migration prone lines. Similar differences in VEGF and ICAM-1 expression were detected in clinical samples from patients with high and low grade gliomas. The migration prone lines displayed increased expression of miR-21 which was also observed in the advanced patient samples. A miR-21 mimic enhanced the migration properties and anti-apoptotic protein expression in the U251 cells. CUR was determined to decrease both miR-21 and anti-apoptotic protein expression. CUR also induced proteins associated with cell death such as LC3-II and other proteins in U251 cells. In contrast, in the migration-prone derivative lines, such increases in proteins associated with cell death were not observed after CUR treatment. These studies point to the importance of miR-21 in migration and survival of glioma cells [111].

CUR treatment of U-87 glioblastoma cells resulted in miR-146A expression which was determined to be involved in the CUR-mediated enhancement of temozolomide cytotoxicity. The combined CUR and temozolomide treatment resulted in enhanced toxicity in U-87 glioblastoma cells. The expression of miR-146a was determined to be necessary for the enhanced toxicity. Overexpression of miR-146a resulted in decreased NF-kappaB activation on temozolomide-treated cells. Suppression of NF-kappaB activity enhanced temozolomide-induced apoptosis. These studies point to a mechanism by which CUR can sensitize glioblastoma cells to temozolmide, induction of miR-146a and suppression of NF-kappaB activity [112].

**Figure 6:**
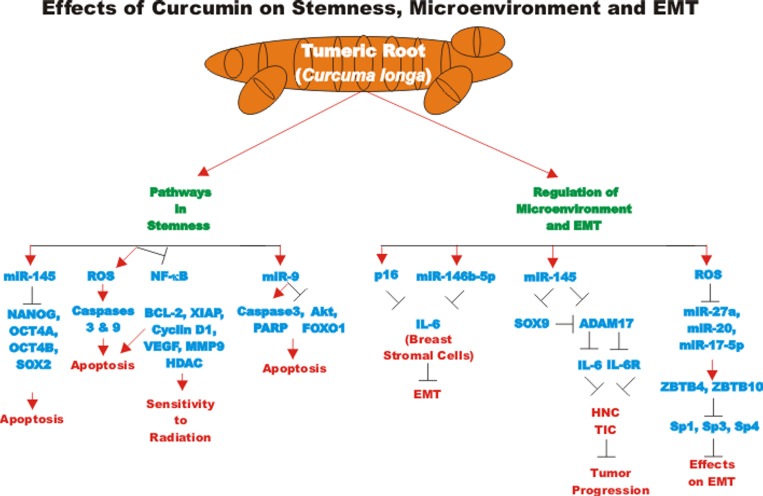
Overview of effect of curcumin on stemness, microenvironment and EMT An overview of the effects of CUR on pathways involving stemness, microenvironment and EMT pathways and the effects of miRs are indicated. Red arrows indicate induction of an event; black closed arrows indicate suppression of an event.

The effects of CUR encapsulated in a nontoxic nanocarrier, called dendrosomes have been examined on U87MG glioblastoma cells. Dendrosomal curcumin increased the expression of miR-145 and decreased the expression of stemness genes including: NANOG, OCT4A, OCT4B1, and SOX2 [113]. A diagram of some of the effects of CUR on pathways involved in stemness is presented in Figure 6.

### Effects of curcumin on breast cancer cells

CUR has been shown to have effects on drug transporter expression in breast cancer cells with CSC properties. CUR could increase the tumoricidal effects of mitomycin C by suppressing ABCG2 expression. In these studies, CUR could also increase the sensitivity of MCF-7 and MDA-MB-231 cells to various chemotherapeutic drugs including: cisplatin, doxorubicin and paclitaxel. The combination of CUR and mitomycin C together prevented the sphere forming capacity of MCF-7 and MDA-MB-231 cells after five passages. These effects were only observed when both drugs were added together. The effects of CUR on the sphere forming capacity of the breast cancer cells were determined to be dependent primarily upon ABCG2 expression [114].

CUR has been shown to affect microtentacles (McTN) that are present in mammospheres with CSC properties. McTN are tubulin-based protrusions present in detached cells. McTN may be involved in cell adhesion and attachment and have been hypothesized to be involved in metastasis. CUR was shown to affect the McTNs and prevented reattachment [115].

Treatment of breast stromal fibroblasts with CUR was determined to increase the level of both p16^INK4A^ mRNA and miR-146b-5p. miR-146b-5p suppressed IL-6 levels in a p16^INK4A^-dependent fashion. This resulted in a reduction of the paracrine pro-carcinogenic effects of the breast stromal fibroblasts which can stimulate EMT in breast cancer cells in a paracrine fashion. miR-146-5p binds a region in the 3′UTR of the IL-6 mRNA and suppresses it function. In contrast, suppression of miR-146b-5p results in activation of breast stromal fibroblasts [116].

Bisphenol A (BPA) is used in the manufacture of plastics such as water bottles. BPA may be important in breast cancer development as it is an endocrine disrupter. The ability of CUR to protect against the detrimental effects of BPA is not clear. BPA can have estrogenic-like effects on ER+ MCF-7 cells and increase their proliferation. CUR was shown to inhibit the effects of BPA on the breast cancer cells. BPA induced the expression of oncogenic miR-19a and miR19b. BPA has effects on the expression of PTEN and pAKT, pMDM2, TP53, and PCNA which are growth promoting. These effects that BPA had were reversed upon CUR treatment [117].

Emodin is a compound present in the root and rhizome of the *Rheum palatum* plant. The effects of emodin and CUR have been examined on breast cancer cells. A synergistic interaction was observed when emodin and CUR were combined in terms of inhibition of cell growth, survival and invasion. miR-34a expression was upregulated by emodin and CUR. miR-34a can decrease the expression of BCL-2 and BMI1 [118].

The effects of CUR on MCF-7 breast cancer cells have been examined. CUR was shown to inhibit BCL-2 expression by increasing miR-15a and miR-16 in MCF-7 cells. Silencing miR-15a and miR-16 increased the expression of BCL-2 expression [119].

### Effects of curcumin on colorectal cancers (CRC)

CUR has been shown to target CRC CSC. The effects of CUR can be enhanced by various anti-cancer agents. CUR may act as a chemosensitizer and target multiple signaling pathways including: WNT/beta-catenin, sonic hedgehog (SHH), NOTCH and PI3K/PTEN/Akt/mTORC and have effects on EMT and regulation of miRs. Cur may influence CRC CSC [120].

CUR has been shown to interact with the CD44 (a receptor for hyaluronic acid) and stimulate apoptosis in CRC CSC. While CUR induced some apoptosis in both CRC cancer cells and CRC CSCs, CUR induced more apoptosis in CRC CSCs. The authors proposed that CUR may interact with CD44+ at the cell membrane and block the entry of glutamine into the cells [121].

The effects of CUR have been recently examined on patient-derived colorectal liver metastases (CRLM). The ability of CUR to augment FOLFOX treatment was determined first in CRC CSC models and then in a phase I dose escalation study. Interestingly treatment with CUR alone and CUR in combination with FOLFOX reduced CRLM sphere forming numbers. CUR also reduced the number of cells with high aldehyde dehydrogenase activity (ALDH(high)/CD133(-) phenotype. CUR was determined to be safe and well-tolerated in combination with FOLFOX chemotherapy in the clinical trial with twelve CRLM patients [122].

Doublecortin-like kinase 1 (DCLK1) is an important kinase in the doublecortin kinase family which have multiple neurological functions and has also been implicated in stem cells. CUR is normally thought to induce apoptosis in cancer cells by multiple mechanisms. Recently, CUR has been determined to promote the survival of DCLK1+ CRC CSCs by inducing autophagy. CUR was determined to reduce the expression of DCLK1/CD44/ALDHA1/LGR5/NANOG in CRC CSC three-dimensional spheroid cultures as well as in tumor xenografts. Surprisingly, CUR also promoted autophagic survival of some DCLK1-positive CSCs. Suppression of DCLK1 induced apoptosis and prevented the pro-survival effects of CUR in the subset. These authors suggested that suppression of DCLK1 may enhance the effects of CUR on certain CRC CSC subsets [123].

**Figure 7:**
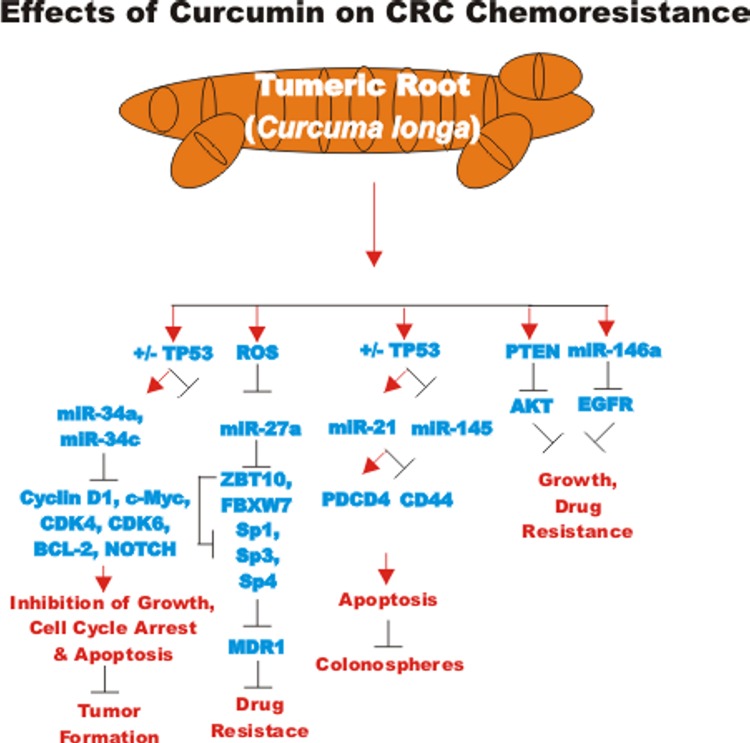
Effects of curcumin on CRC chemoresistance An overview of the effects of CUR on CRC chemoresistance and the effects of miRs are indicated. Red arrows indicate induction of an event; black closed arrows indicate suppression of an event.

CDF can induce miR-34 which is normally silenced in CRC. miR-34a and miR-34c were determined to be down-regulated in CRC patient samples compared to normal colonic mucosa. The decreased expression of these miRs is believed to be due to promoter hypermethylation as treatment with 5-AzaC induces their expression. CDF also induced the expression of these miRs which suppressed NOTCH1 [124]. A diagram of some of the effects of CUR on suppression of CRC chemoresistance is presented in Figure 7.

The effects of 5-FU and CUR on DNA mismatch repair (MMR) status in CRC CSCs have been examined. These studies employed 3D cultures of HCT116, HCT116+ch3 (+ chromosome 3) and 5FU-resistant clones cultured in the presence and absence of CUR. These studies demonstrated that pre-treatment of the resistant cells with CUR increased the effects of 5FU and resulted in the disintegration of colonospheres and apoptosis. A combination of CUR and 5FU was determined to affect MMR-deficient CRC cells more effectively than MMR-competent cells [125].

The effects of CUR and 3 acetyl-11-keto-β-boswellic acid (AKBA) have been investigated on CRC cells. The expression of both miR-27a and miR-34a were both altered in CRC cells after CUR or AKBA treatment [126]. ROS have been proposed to be responsible for the ability of curcuminoids to suppress drug resistance of SW-480 CRC cells. This is believed to occur by disruption of miR-27a/zinc finger and BTB domain containing 10 (ZBTB10) signaling. Curcuminoids were prepared in this study by HPLC. Their effects were examined on CRC cell lines SW-480 and HT-29 cells and normal CCD-18Co colon fibroblast cells. The curcuminoids were shown to affect the CRC HT-29 and SW-480 cell lines but not the normal colon fibroblast cells. The curcuminoids enhanced the effects of 5FU on the drug resistant cells by suppression of MDR1. CUR has been shown to down-regulate the expression of miR-27a in CRC cells. This may occur by CUR inducing ROS which results in suppression of specificity protein expression (SP1, SP3 and SP4) as well as miR-27a. The ZBTB10 gene is normally a target of miR-27a and upon downregulation of miR-27a by CUR, increased expression of ZBTB10 occurred. ZBTB10 is a transcriptional repressor of SP expression. CUR suppressed SP1 and SP3 expression which in turn led to decreased MDR1 expression and decreased drug resistance [127–129].

FBXW7 (F-box and WD repeat domain-containing 7) is a tumor suppressor which is an E3-ubiquitin ligase involved in the ubiquitination of many growth related proteins. FBXW7 is regulated by many molecules *e.g*., TP53 and miRs *e.g*., miR-27a. FBXW7 is frequently mutated in CRC [130]. Deletions of TP53 and FBXW7 may be involved in the progression of CRC from adenoma to carcinoma at least in animal models [131]. While CUR can exert some cytotoxic effects on TP53- CRC, the combination of CUR and AKBA showed greater effects in TP53- CRC and this was determined to involve FBXW7. Treatment of TP53- cells with CUR and AKBA resulted in increased FBXW7 expression as well as downregulation of its target genes [131].

CRC colonospheres have been determined to have elevated miR-21 but decreased PTEN expression. CDF downregulated the expression of miR-21 in chemo-resistant HCT116 and HT-29 cells. Moreover, PTEN levels were restored and inhibited activated Akt expression after CDF treatment [132].

The transcriptional start sites of the miR-21 gene were identified in Rko and HCT116 cells. PMA was shown to induce the expression of miR-21 via AP-1 sites in the promoter region. CUR suppressed the binding to AP-1 to the miR-21 promoter region. The expression of the tumor suppressor PDCD4 was induced as it is a target of miR-21[133].

Certain chemotherapy-resistant CRC cells have elevated levels of miR-21. These chemoresistant cells are enriched with cells with CSC phenotypes. One marker associated with CRC CSCs is CD44. Suppression of miR-21 decreased the presence of cells with the CSC phenotype in HCT-116 and HT-29 CRC cells. The effects of CDF, FLUFOX and the CDF + FULFOX combination were examined on these cells. Induction of differentiation increased the sensitivity of the CRC CSC to the above-mentioned treatment. The growth inhibition properties were greater in the CDF or CDF + FLUFOX treated cells. Treatment of differentiating cells with CDF or CDF + FLUFOX combination reduced the expression of CD44 and EGFR [134].

### Effects of curcumin on cervical cancer

CUR and stattic both inhibit STAT3 in papillomavirus-induced cervical cancer SiHa cells. Reduction of STAT3 expression resulted in a decrease in miR-21 expression. In contrast, specific depletion of miR-21 resulted in an increase in PTEN levels which is a negative regulator of STAT3. Restoration of Let-7a also resulted in a reduction of STAT3 levels. Suppression of the papillomavirus E6 activity resulted in increased Let-7a but decreased miR-21 levels and led to increased PTEN and decreased STAT3 levels. These studies point to the interactions between CUR, E6, miR-21, Let-7a, PTEN and STAT3 in palillomavirus-induced cervical carcinogenesis [135].

### Effects of curcumin on esophageal cancer

CUR inhibits the growth of esophageal cancer cells by suppression of NOTCH signaling. CUR induced apoptosis in the esophageal cells which was associated with caspase-3 activation, an increase in the ratio of BAX/BCL-2 and a decrease in cyclin D1 levels. CUR also decreased sphere formation in the esophageal cells, suppressed NOTCH activation and JAGGED1 and HES expression. The reduction of NOTCH expression was associated with decreased gamma-secretase complex activity. CUR treatment decreased the expression of miR-21 and miR-34a. In contrast, curcumin treatment upregulated tumor suppressor let-7a miRNA [136].

### Effects of curcumin on head and neck cancers

The ability of CDF, packaged in liposomes, to inhibit HNSCC growth has been determined recently on cisplatin-resistant HNSCC cell lines. The liposomal-CDF was determined to inhibit proliferation, tumor formation and CD44 expression in the cisplatin-resistant CCL-23R and UM-SCC-1R HNSCC lines. These studies suggest that liposomal-CDF may be an approach to deliver CDF to cisplatin-resistant HNSCC tumors [137].

CUR treatment has been shown to induce miR-145 promoter activity in head and neck squamous cell carcinomas (HNC). This resulted in decreased SOX9 expression. SOX9 can normally induce ADAM metallopeptidase domain 17 (ADAM17) expression. Thus, the induction of miR-145 by CUR-inhibited SOX9 and ADAM17 expression which are important in the generation of HNC CSCs [138].

### Effects of curcumin on HCC

**Figure 8:**
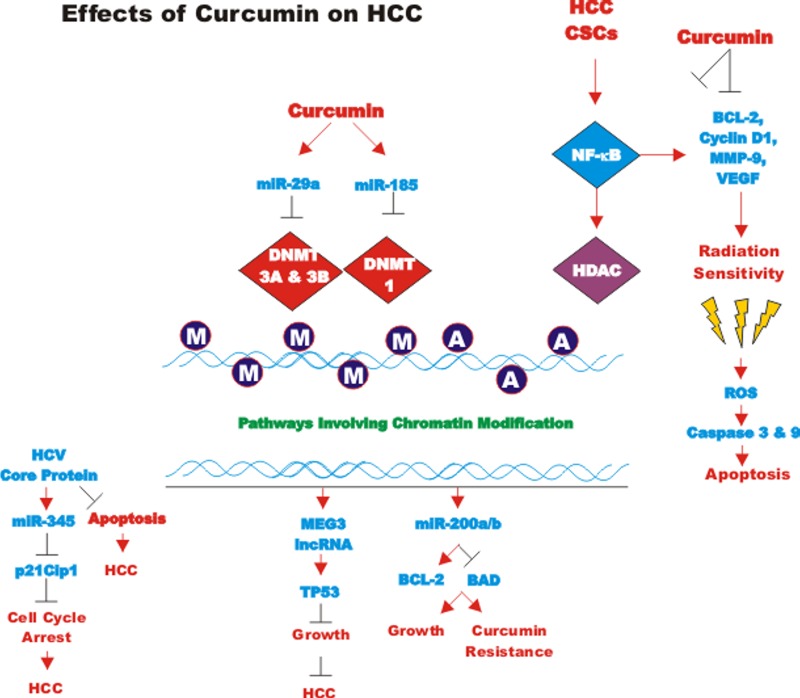
Effects of curcumin on HCC An overview of the effects of CUR on HCC is presented. M = methylated residue, A = acetlylated residue. Red arrows indicate induction of an event; black closed arrows indicate suppression of an event.

CUR has been shown to produce significant growth inhibitory and apoptotic effects in human HCC cells that expressed constitutively-activated NF-kappaB. This was determined to be due, in part, to ROS generation and mainly dependent on activation of caspase-9 and -3. In addition, CUR used in combination with cisplatin resulted in a synergistic cytotoxic effect, while the effects were additive or sub-additive in combination with doxorubicin [139]. A diagram of the effects of CUR on HCC is presented in Figure 8.

NF-kappaB signaling is one of the key pathways frequently activated in liver CSCs [140]. It was recently shown that treatment of CUR-sensitive HCC CSCs with CUR selectively reduces the number of CSCs as evidenced by a reduced side positive (SP) staining, down-regulation of CSC markers, decrease in sphere formation, and suppressed tumorigenicity. Conversely, the CUR-resistant cells show a paradoxical increase in the proliferation and expression of CSC markers. In terms of the molecular mechanism, an important component of the CUR-mediated CSC-depletion was attributed to NF-kappaB-mediated HDAC inhibition. Consistently, the combination CUR and trichostatine, an inhibitor of class I/II HDAC, has been shown to sensitize resistant cells to CUR [141].

Some of the effects of CUR treatment are inhibition of NF-κB activity and downstream effector proteins, including: VEGF, MMP-9, XIAP, BCL-2 and Cyclin-D1. This was demonstrated to sensitize the HCC cells to radiation [142]

Piperine is an alkaloid found in the seeds of black pepper (*Piper nigrum*) and is known to enhance the bioavailability of several therapeutic agents, including CUR [143]. Recently, it was demonstrated that co-administration of CUR with piperine resulted in a significant protection against diethylnitrosamine (DENA)-induced hepatocellular carcinoma in rats in comparison with CUR by itself [144].

CUR inhibits HIF-1 in certain HCC cell lines and *in vivo* studies with tumor xenografts This was shown to occur by CUR inducing the degradation of the aryl hydrocarbon receptor nuclear translocator (ARNT). HIF-1 and ARNT normally form a complex and CUR prevented this complex from forming. CUR was demonstrated to induce the proteasomal degradation of ARNT via oxidation and ubiquitination [145]. CUR also inhibited EMT by suppressing HIF-1alpha activity in HepG2 cells [146].

Phthalates are chemicals added to plastics and they are referred to as plasticizers [147]. They serve to increase the durability, flexibility, longevity and transparency of plastics. They also may have adverse effects on human health as they may promote cancer development. The bis(2-ethylhexyl) phthalate has recently been shown to stimulate migration, invasion and EMT in HCC. bis(2-ethylhexyl) phthalate also increased the presence of HCC CSCs, stemness and growth and metastasis in animal studies. CUR was shown to suppress these properties of bis(2-ethylhexyl) phthalate through inhibition of the aryl hydrocarbon receptor/ERK/SK1/S1P3 signaling pathway [148].

MEG3 encodes a lncRNA which is a tumor suppressor. There is decreased expression of the MEG3 lncRNA in HCCs. The decreased expression of MEG3 is believed to be due to promoter methylation. CUR has been shown to alter the DNA methylation status. The effects of CUR on DNA methylation may be carried out by DNMT1, DNMT3A and DNMT3B. miR-29a was determined to inhibit DNMT3A and DNMT3B and miR-185 was shown to affect DNMT1. When DNMT1, DNMT3A and DNMT3B were decreased by dendrosomal CUR treatment, increased expression of MEG3 occurred which was due to elevated expression of miR-29a and miR-185. Thus, dendrosomal curcumin can result in DNA hypomethylation and expression of previously silenced tumor suppression genes that may be important in HCC [149, 150].

The expression of miR-200 family members was examined in HepG2 and HepJ5 HCC cell lines. HepG2 cells expressed higher levels of miR-200a/b and were more resistant to CUR than HepJ5 cells. Overexpression of miR-200a/b conferred resistance to CUR in HepJ5 cells [151]. Thus, targeting miR-200a/b may overcome CUR-resistance.

The core protein of HCV has been shown to inhibit the apoptosis normally induced by CUR in Huh7 HCC cells. The core protein of HCV normally induces miR-345 which targets and suppresses p21^Cip-1^ [152].

CUR has a poor solubility in aqueous enviroment, and consequently it has a low bioavailability and therefore low concentrations at the target sites. To overcome these limitations, various types of nanoparticles have been developed to delivery CUR to targets either alone or in combination with other drugs, such as sorafenib and doxorubicin [153–155]. This approach has provided a promising strategy to enhance CUR antitumor effects on HCC *in vitro* and *in vivo*.

### Effects of curcumin on leukemia and lymphoma

Malignant T cells display decreased expression of miR-22 in comparison to normal T cells. STAT5 has been shown to bind to the promoter region of miR-22. Suppression of the Jak3/STAT3/STAT5 resulted in an increase in pri-miR-22 and subsequently miR-22. Histone deacetylase inhibitors (HDACi) also stimulate miR-22 expression. CUR inhibited Jak-3 activity and stimulated miR-22 expression. In these studies, miR-22 was determined to have many effects such as inhibition of: cyclin dependent kinase 2 (*CDK2*), histone deacetylase 6 (*HDAC6*), MYC associated factor X (*MAX*), MYC binding protein (*MYCBP*), nuclear receptor coactivator 1 (*NCOA1*), and *PTEN*. These genes have previously been implicated in cutaneous T-cell lymphoma (CTCL) [156].

Histone modifying enzymes have been shown to be regulated by CUR and miR-34. Histone modifying enzymes play key roles in regulation of gene expression because they regulate the accessibility of promoter regions present in genes contained in chromatin to transcription factors. The regulation of the histone modification genes was examined in pediatric acute lymphoblastic leukemia (ALL). The expression profile of the histone modification genes was different from normal control samples. The histone deacetylases HDAC2 and PAK1 were upregulated in the ALL samples while other genes including PRMT2 and the putative tumor suppressor EP300 were downregulated. Ingenuity Pathway Analysis revealed that CUR and miR-34 could be regulators of the histone-modifying genes in ALL [157].

CUR treatment has been shown to induce promoter demethylation of the p15 gene (*CDKN2B* cyclin dependent kinase inhibitor 2B, pINK4B) in RAJI ALL cells. This resulted in the induction of apoptosis in these cells [158].

miR-368 is involved in regulation of DNA damage repair. miR-368 is upregulated in terminally-differentiated cells. miR-368 is involved in regulating the sensitivity of leukemia cells to chemotherapeutic drugs such as cisplatin. The structural maintenance of chromosome 1A (SMC1A) is a target of miR-658. Increased expression of miR-658 elevated the sensitivity of certain leukemia cells to cisplatin. miR-638 was demonstrated to inhibit the recruitment of H2AX to DNA break sites [159].

### Effects of curcumin on BCR-ABL-transformed leukemia models

The ability of a CUR derivative (C817) to inhibit the growth of imatinib-resistant chronic myeloid leukemia (CML) cell lines has been determined. C817 could suppress the proliferation of imatinib-resistant murine 32D cells which had mutant BCR-ABL genes (nucleotide binding P-loop mutants Q252H, Y253F, and imatinib contact residue mutant T315I) or imatinib-resistant human erythroleukemia K562/G01 cells with amplified WT BCR-ABL genes. C817 was determined to inhibit BCR-ABL kinase activity in imatinib-resistant cells with mutant and WT BCR-ABL in the low nanomolar range. C817 inhibited BCR-ABL, STAT-5 and CRKL phosphorylation in imatinib-resistant cells. C817 may also suppress leukemia progenitor/stem cells [160].

CUR also has been shown to inhibit CML growth by exosomal disposal of miR-21. Exosomes can contain miRs and proteins and other cellular components which can be released by cells and influence the proliferation of other cells. CUR treatment has been shown to result in activation of PTEN, which is a target of miR-21. In contrast, CUR treatment resulted in a decrease of VEGF and activated Akt. The presence of miR-21 in exosomes from K562 and LAMA84 cells after CUR treatment was determined. CUR treatment also resulted in an increase of miR-196b which lead to decreased levels of BCR-ABL expression. The size of CML xenografts in mice was smaller in CUR-treated SCID mice than in controls. Exosomes from CUR-treated cells were enriched with miR-21 compared to controls. These results indicate a mechanism by which CUR may elicit anti-tumor effects in CML [161].

CUR has been shown to induce the expression of miR-21 in CML. miR-21 has been detected in tumor derived exosomes and was shown to affect the angiogenic phenotype. The miR-21 present in the CML-derived exosomes was shuttled into endothelial cells. When HUVEC cells were exposed to CML-derived exosomes, increases in IL-8 and vascular cell adhesion protein 1 (VCAM1) were observed. However, in HUVEC cells treated with exosomes derived from CML cells that had been exposed to curcumin, these increases in IL-8 and VCAM1 were not detected and reduced RAS homolog family member B (RHOB) expression and mobility was observed. Thus, the CUR-derived exosomes were suppressing angiogenesis [162].

### Effects of curcumin on lung cancer cells

CUR has been determined to suppress the tumor sphere formation capacity in lung cancer H460 cells by suppressing the JAK2/STAT3 signaling pathway. CUR also suppressed lung cancer xenograft formation in mouse studies [163].

CUR is postulated to exert its effects on cancer via multiple mechanisms including more than one mechanism at the same time. It may have anti-angiogenic, anti-oxidant, immunomodulatory and pro-apoptotic effects. Recently in lung cancer cells, CUR has been shown to alter the expression of enhancer of zeste homolog 2 (EZH2), by both transcriptional and post-transcriptional mechanisms. CUR also suppressed EZH2 expression by induction of miR-let 7c and miR-101. The expression of NOTCH1 was inhibited upon EZH2 suppression [164].

CUR has been shown to activate the TP53/miR-192-5p/miR-215/XIAP pathway in NSCLC. CUR activated both miR-192-5p and miR-215 in TP53 WT A427 cells. miR-192-5p and miR-215 functioned as tumor suppressors in these cells. Conditional knockdown of TP53 in *TP53* WT H460, A427 and A549 cells abrogated the effects of CUR. Expression of WT TP53 in TP53-mutant H1299 resulted in miR-192-5p and miR-215 expression after CUR treatment. The effects of CUR were shown to be dependent on miR-192-5p and miR-215 expression. These studies revealed that the X-linked inhibitor of apoptosis (XIAP) was a novel transcription target of miR-192-5p and miR-215 in non-small cell lung cancer [165].

CUR can also suppress proliferation and induce apoptosis in NSCLC via suppression of the PI3K/PTEN/Akt pathway. This was shown to be mediated by CUR inducing miR-192-5b expression in A549 cells. Increased miR-192-5b expression in A549 cells resulted in decreased proliferation and increased apoptosis while suppression of miR-192-5b had the opposite effects. CUR increased miR-192-5p expression while it decreased PI3K/PTEN/Akt activity. miR-192-5p mimic could increase the effects of CUR [166].

CDF elicits enhanced anti-cancer activity. CDF inhibited MMP2 expression more effectively in A549 and H1299 cells than CUR. CDF increased the expression of miR-874 which targets MMP-2 [167].

The effects of CUR on miR-21 expression in NSCLC A549 cells were determined. CUR treatment resulted in suppression of miR-21 and an increase in PTEN which is a target of miR-21 [168].

CUR has been shown to increase the effectiveness of chemotherapy on the CD166+/EpCAM+CSC subpopulation in two NSCLC cell lines (A549 and H2170). This was ascertained by determining the extent of cisplatin-induced apoptosis and inhibition of metastasis after CUR treatment. CUR decreased Cyclin D1 expression and increased p21^Cip1^ expression. CUR also stimulated the intrinsic apoptotic pathway as APAF1 and Caspase-9 were activated in the CD166+/EpCAM+ CSC subpopulation of A549 cells. These studies have revealed that CUR may enhance the effects of cisplatin on NSCLC CSCs [169].

The effects of CUR on A549 lung cancer cells were further examined. miR-186 was determined to be down-regulated by CUR treatment of A549 cells. Caspase-10 was determined to be a target of miR-186 [170]. In these studies, with miR-186 and lung cancer, miR-186 appears to have promoted growth and suppression of miR-186 results in apoptosis and drug resistance. CUR appears to decrease miR-186 expression.

The effects of CUR have been examined on multidrug-resistant human lung adenocarcinoma A549/DDP cells. CUR treatment also decreased the expression of miR-186 in these cells. Overexpression of miR-186 suppressed the effects of CUR on the drug-resistant cells. Treatment of the cells with a miR-186 inhibitor induced apoptosis of the cells [171].

miR-346 is an oncomir for NSCLC. It can affect cell growth and metastasis. The xeroderma pigmentosum complementation group C (XPC) gene is involved in nucleoside excision repair and is a target for miR-346 [172].

### Effects of curcumin on melanoma

Diphenyl difluoroketone (EF24) is another CUR analog. EF24 can arrest cell cycle progression, induce apoptosis, inhibit mobility and EMT in melanoma cells and prevent their metastasis *in vivo*. EF24 can induce miR-33b which binds and inhibits high-mobility group AT-hook 2 (HMGA2) expression in Lu1205 and A375 melanoma cells. HMGA2 is a protein which may be present in the enhanceosome and is a transcriptional regulatory factor. HMGA2 may stimulate the expression of genes by altering chromatin structure. EF24 treatment resulted in increased E-cadherin and decreased N-cadherin, vimentin and STAT3 phosphorylation [173].

The effects of a diet consisting of 4% CUR on mice injected with B78H1 murine melanoma cells were determined. Mice fed the diet containing 4% CUR had reduced growth of the melanoma compared to the control mice. RNA analysis indicated that miR-205 was elevated over 100-times in the mice fed the CUR diet. BCL-2 and PCNA were down regulated in the mice fed the CUR diet [174].

### Effects of curcumin on nasopharyngeal carcinoma

CUR inhibited the expression of miR-125-5p, miR-574-3p and miR-210 in undifferentiated nasopharyngeal carcinoma (NPC). In contrast, forced expression of miR-125-5p stimulated proliferation, migration and invasion of HONE1 cells. miR-125-5p was determined to be detected at higher levels in NPC than in healthy controls. miR-125-5p inhibited TP53 expression. CUR was determined to inhibit miR-125-5p but increase TP53 expression [175].

The Ewing Sarcoma associated transcript (EWSAT1) is a long non-coding RNA. It is expressed in NPC. Recently EWSAT1 was determined to be upregulated in NPC tissues and associated with poorer survival. EWSAT1 upregulated the expression of Cyclin D1 by acting as a competitive “sponge” for miR-326/330-p5 clusters. Thus, by sponging miR-326/330-p5, Cyclin D was detected at higher levels [176].

### Effects of curcumin on oral cancer

CUR regulated the expression of miR-9 in oral squamous cell carcinoma (OSCC). The levels of miR-9 were significantly lower in clinical OSCC patient samples than in adjacent non-tumor specimens. Suppression of miR-9 by anti-miR-9 oligonucleotides prevented the effects of CUR on growth suppression and re-activated WNT/beta-catenin signaling [177].

miR-31 is upregulated and acts as an oncogene in OSCC. EGF activation of EGFR signaling in OSCC results in the increased expression of miR-31, miR-181b and miR-222 expression. Inhibition of AKT or knockdown of C/EBPbeta suppressed miR-31 up-regulation. CUR treatment inhibited AKT activation, upregulation of C/EBPbeta and miR-31 [178].

### Effects of curcumin on osteosarcoma

**Figure 9:**
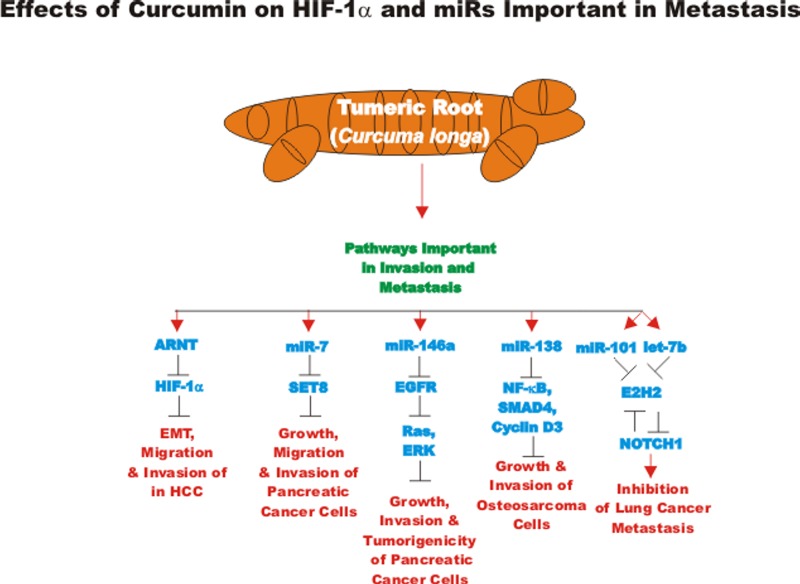
**Overview of effects of curcumin on HIF-1alpha, miRs and metastasis.** An overview of the effects of CUR on pathways involving migration, invasion and metastatic pathways are indicated. Red arrows indicate induction of an event; black closed arrows indicate suppression of an event.

CUR has also been shown to suppress proliferation and invasion of the human osteosarcoma cell line MG-63. This was shown to occur by CUR inducing miR-138 expression. mRNAs encoding SMAD4, NF-kappaB p65 and Cyclin D3 are targets of miR-138 and they were suppressed by miR-138 after CUR treatment. In contrast, inhibition of miR-138 was shown to increase the expression of SMAD4, NF-kappaB p65 and Cyclin D3. A diagram of the effects of CUR on invasion and metastasis is presented in Figure 9 [179].

### Effects of curcumin on ovarian cancer

CUR treatment has been determined to result in an increase in miR-9 expression in SKOV3 ovarian cancer cells. Inhibition of miR-9 expression prevented the growth inhibitory effects of CUR while overexpression of miR-9 increased cleavage of caspase-3, PARP and apoptosis. Both CUR treatment and overexpression of miR-9 resulted in decreases in phosphorylation of Akt and FOXO1 [180]. Recently the effects of HCC formulated in nanoparticle has been shown to be more effective in suppressing the proliferation of certain ovarian cancer cell lines than administration of CUR by itself [181].

### Effects of curcumin on pancreatic cancer cells

CD44 has been implicated in the MDR of pancreatic cancer cells. Approaches to target CD44 may eventually be appropriate for treatment of drug-resistant pancreatic cancers. Hyaluronic acid (HA) targeting may be an approach to suppress drug resistant pancreatic cancers. Recently, the HA conjugate of copoly(styrene maleic acid) (HA-SMA) has been used to make nanomicelles with CDF and is referred to as HA-SMA-CDF. The effects of these HA-SMA-CDF nanomicells on the MIA-PaCa-2 and AsPC1 pancreatic cancer cells have been examined. The HA-SMA-CDF nanomicells were shown to elicit stronger anti-cancer responses against CD44+/CD133+/EpCAM+ CSCs compared with CD44-/CD133-/EpCAM- cells. The HA-SMA-CDF nanomicelles inhibited NF-kappaB expression which is important in proliferation and invasion and other properties associated with tumor development [182].

miR-7 has been shown to be regulated by CUR in pancreatic cancer cells. CUR suppressed growth of pancreatic cancer cells. The histone lysine methyltransferase SET8 is a target of miR-7. CUR was determined to increase miR-7 and decrease SET8 expression [183].

The expression of miR-146a has been examined in pancreatic cancer patient samples. miR-146a was detected at lower levels in approximately 80% of pancreatic cancer samples as opposed to non-cancerous tissues. One of the targets of miR-146a is EGFR. Treatment with the modified CUR, CDF, resulted in the re-expression of miR-146a which inhibited EGFR expression. Re-expression of miR-146a or treatment with CDF inhibited tumor xenografts as well as reduced EGFR, ERK1, ERK2, and KRAS expression. Knockdown of miR-146a in the pancreatic AsPC-1 cell line resulted in increased EGFR expression and enhanced clonogenic growth. Suppression of EGFR or ectopic expression of pre-miR-146a inhibited the invasive properties of the cells [184].

CDF induced the expression let-7a, b, c, d miRs, miR-26a, miR-101, miR-146a, and miR-200b, c in pancreatic cancer. These miRs are normally silenced in pancreatic cancer. In contrast, CDF repressed the expression of the histone methyltransferase EZH2 and EpCAM. In an orthotopic pancreatic tumor model, CDF also inhibited the expression of EZH2, NOTCH-1, CD44, EpCAM, and NANOG. In *in vitro* cell lines assays, CDF increased expression of let-7, miR-26a, and miR-101 [185].

The expression of the miR-200 family, PTEN and membrane type-1 matrix metalloproteinase (MT1-MMP) has been examined in pancreatic cancer cell lines. Loss of expression of miR-200a, miR-200b and miR-200c was associated with decreased PTEN expression and increased expression of MT1-MMP. The synthetic analog CDF induced the re-expression of the miRs and PTEN while MT1-MMP was decreased in BxPC-3, MIA-PaCa-2 and gemcitabine-resistant MIA-PaCa-2-GR cells as was as cell growth [186].

CDF could inhibit sphere formation capacity of pancreatic cancer cell lines and inhibit the expression of the CSC markers CD44 and EpCAM. CDF also inhibited xenograft formation and decreased NF-kappaB activity, miR-21 and COX levels and increased PTEN and miR-200 levels [187].

Gemcitabine is a nucleoside analogue which is used in pancreatic and other cancer therapies. CUR inhibits tumor growth but its effects are limited due to its poor bioavailability. The effects of CDF have been examined in gemcitabine-sensitive and gemcitabine-resistant pancreatic cancer cell lines. Downregulation of AKT, COX2, prostaglandin E2 (PEG2), VEGF, and NF-kappa-B activity was observed after CDF treatment. The effects of CDF on miR-200 and miR-21 were analyzed. CDF increased the expression of miR-200 and decreased the expression of miR-21 which resulted in increased PTEN protein levels [188].

The expression of miR-221 has been shown to be elevated in pancreatic cancer cells compared to normal pancreatic duct epithelial and tissue. In addition, pancreatic cancer patients with high miR-221 expression had a shorter survival in comparison with patients which had lower miR-221 expression. Inhibition of miR-221 resulted in a decrease in proliferation and increased expression of: PTEN, p27^Kip1^, p57^Kip2^ and PUMA which are likely targets of miR-221. The effects of isoflavone mixture (G2535), formulated 3,3′-diindolylmethane (BR-DIM), and CDF were examined on MIA-PaCa-2 and PANC21 cells. Treatment with these compounds decreased the levels of miR-221 and increased the levels of: PTEN, p27^Kip1^, p57^Kip2^ and PUMA [189].

### Effects of curcumin on prostate cancer cells

CUR has been shown to have some effects on signaling pathways implicated in prostate cancer [190]. CUR has been shown to have effects on cancer associated fibroblasts (CAFs). CAFs are important in regulation of EMT in tumor cells. CAFs are also important in the induction of stem cell characteristics. CAFs were determined to induce EMT and invasion through a monoamine oxidase A (MAOA)/mTOR/HIF-1alpha signaling pathway in prostate cancer cells. This pathway induced ROS which resulted in the migratory and aggressive characteristics of the prostate cancer cells. The CAFs also increased CXCR4 and IL-6R expression in the prostate cancer cells. CUR was shown to inhibit all these processes [191].

The modified CUR EF24 has been shown to target the expression of miR-21 and NF-kappaB in DU145 prostate cancer and B16 murine melanoma cells. EF24 also increased the expression of various miR-21 target genes including PTEN and programmed cell death 4 (PDCD4) a gene which inhibits neoplastic transformation [192].

CUR has been shown to inhibit prostate cancer growth. CUR induced the accumulation of cyclin-dependent kinase inhibitor 1A (CDKN1A = p21^Cip-1^) protein but not its mRNA in a dose-dependent fashion suggesting that CUR elicited effects at the post-transcriptional level, *e.g*., at the translational level. It was determined that miR-208 was specifically inhibited by CUR. Furthermore, miR-208 specifically bound the 3′untranslated region (3′UTR) of CDKN1A mRNA. Overexpression of miR-208 in prostate cancer cells prevented the inhibitory effects that CUR has on proliferation [193].

CUR can also induce the demethylation of the Neurog1 gene which is involved in preventing growth of the LNCaP prostate cancer cell line [194]. CUR can also induce the demethylation of the nuclear factor erythroid-2 (NF-E2) related factor-2 (NRT2) gene which in turn activates (NQO1), heme oxygenase-1 (HO1) and an antioxidant stress pathway which can prevent growth in mouse TRAMP-C1 prostate cancer cells [195].

CUR can have potent effects on cancer associated fibroblasts (CAFs) which are important in prostate cancer progression. CAFs were shown to stimulate EMT and invasion in prostate cancer cells EMT. This was mediated by a monoamine oxidase A (MAOA)/mammalian target of rapamycin (mTOR)/HIF-1alpha signaling pathway. This pathway also involves ROS which induces the prostate cancer migration. CAFs were determined to be responsible for the increase in CXC chemokine receptor 4 (CXCR4) and interleukin-6 (IL-6) receptor. CUR treatment inhibited these pathways as well as the invasion and EMT of the prostate cancer cells that was normally induced by the CAFs [196].

The effects of novel poly(lactic-co-glycolic acid)-CUR nanoparticles (PLGA-CUR NPs) on prostate cancer cells were determined. The PLGA-CUR NPs were determined to internalize in prostate cancer. Upon internalization, they release bioactive CUR into the cells and inhibited colony formation and tumor regression better than regularly delivered (free) CUR. The PLGA-CUR NPs were determined to inhibit beta-catenin and AR expression in cells. PLGA-CUR NPs also suppressed Akt and STAT3 phosphorylation which resulted in the induction of apoptosis. Part of the suppression of apoptosis was determined to be by inhibition of MCL-1 and BCL-X_L_ expression and induction of PARP cleavage. PLGA-CUR NPs also resulted in decreased miR-21 and increased miR-205 expression [197].

### Effects of curcumin on hypoxia, HIF and ROS

Hypoxia is important in many key events involved in cancer development including cell survival, tumor invasion, angiogenesis and metastasis. HIF overexpression induced by hypoxia is linked with therapeutic-resistance. Hypoxia and HIF are important in EMT, CSCs and inflammation, all contributing factors for therapeutic resistance. In studies with pancreatic cancer cells, hypoxia and HIF have been shown to be important in the expression of IL-6, miR-21, miR-210 and VEGF as well as the CSC-related factors: NANOG, OCT-4 and EZH2. The expression of these proteins can be inhibited by treatment with CDF under hypoxic conditions. These studies were further elucidated with an orthotopic pancreatic model and similar results were obtained [198].

Other studies have shown that CUR and synthetic analogs can induce ROS and decrease SP factors by the induction of specific miRs in CRC cells. The effects of cyclohexanone and piperidine analogs of CUR were examined in this study. The IC_50_ ranged from 10 μM for Cur to 700 nM for the most active synthetic piperidine analog RL197. CUR and RL197 inhibited the expression of the SP transcription factors and genes they regulated such as EGFR, c-MET, survivin, BCL-2, Cyclin D1 and both p65 and p50 subunits of NF-kappaB. This was determined to occur by CUR and RL197 inducing ROS. ROS induced the expression of ZBTB4 and ZBTB10 which inhibited the expression of the SP proteins and various miRs including: miR-27a, miR-20a, miR-17-5p which normally regulate ZBTB4 and ZBTB10 [199].

### Effects of curcumin on WNT, NOTCH and HH signaling pathways

CUR suppresses the release of cytokines such as: IL-1, IL-6 and IL-8 which can stimulate the generation of CSCs. In addition, CUR suppresses various signaling pathways including WNT, NOTCH, HH and FAK. While curcumin has effects against CSC, it had less toxicity against normal stem cells [200].

### Curcumin—problems with biological extracts

Most sources of CUR are biological extracts and not homogenous preparations of CUR. Numerous other “nutraceuticals” may be present in these extracts which makes identification of a specific biological properties difficult to precise ascribe to CUR. Recently the ability of CUR to acts as panassay interference compounds (PAINS) and an invalid metabolic panaceas (IMPS) compound has been discussed. CUR has been reported to have poor pharmacokinetic (PK) and pharmocodynamic properties (PD). CUR has been suggested to lack the properties required to be a good drug candidate [201].

Clinical trials have so far not revealed that CUR is effective clinically although some of the problems with CUR may be due to the low solubility of CUR and its rapid turnover. This may have resulted from the methods used to deliver CUR to the patient as well as the duration of patient treatments. Furthermore, some of the patients selected for the clinical trial may have had advanced diseases which could preclude the evaluation of the effectiveness of treatment with a drug or natural products which may take years to elicit demonstrable biological effects in humans. However, the presence of the additional components in the CUR extract may provide certain health benefits which may be difficult to biochemically and medically evaluate. More effective means to deliver CUR to patients may be achieved by more novel delivery approaches such as nanoparticles.

### Effects of berberine, a plant derived compound which induces many effects

**Figure 10:**
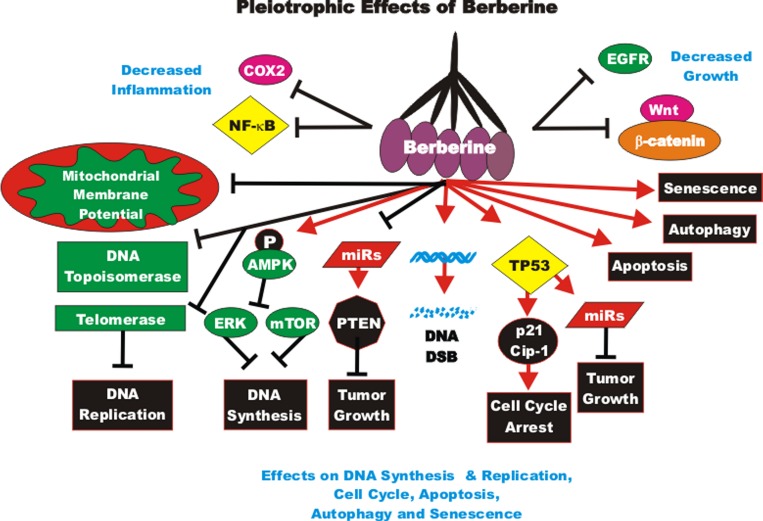
Pleiotropic effects of berberine on signaling pathways involved in cell growth BBRs can induce many pathways which may result in suppression of cell growth, induction of apoptosis, autophagy, senescence, DNA double strand breaks or inhibition of cell cycle progression and DNA replication. These events are indicated by red arrows. In addition, BBR treatment can result in the suppression of many important proteins leading to decreased growth, decreased DNA synthesis, mitochondrial membrane potential, cell cycle arrest and inflammation indicated in black closed arrows.

BBR is an isoquinoline quaternary alkaloid (a 5,6-dihydrodibenzo[a,g]quinolizinium derivative) which has been used in traditional Chinese and Indian medicine for centuries [202, 203]. BBRs are derived from plants such as: *Berberis aetnensis C. Presl., Berberis aristata, Berberis vulgaris, Coptis chinensis, Coptis japonica, Coptis rhizome, Hydrastis canadensis, Phellondendron amurense* and *Tinosora cordifolia*. A diagram illustrating some of the effects of BBR on various biological processes is present in Figure 10. BBR has even been isolated from plants that grow in the vicinity of Mt. Etna in Sicily [204].

BBR, like CUR and RES, is a dietary supplement and can be purchased over the counter at many different types of stores. BBR has been used in treatment of patients with various conditions/diseases including: abdominal pain, coronary artery disease, diabetes, diarrhea, fatty liver disease, gastroenteritis, hyperlipidemia, hypertension, metabolic syndrome, neurodegeneration, obesity, polycystic ovary syndrome and may be used eventually to treat certain cancers. BBR has documented to have anti-diabetic, anti-inflammatory and anti-microbial (both anti-bacterial and anti-fungal) properties. BBRs can inhibit IL-6, TNF-alpha, monocyte chemo-attractant protein 1 (MCP1) and COX-2 production and expression. BBRs can also effect prostaglandin E2 (PGE2) and decrease the expression of key genes involved in metastasis including: MMP2 and MMP9. Some of these effects may be mediated by the Raf/MEK/ERK and NF-kappaB pathways [205, 206].

### Effects of berberine on DNA structure and replication

BBR induces double strand DNA breaks and has similar effects as ionizing radiation [207]. In some cell types, this response has been reported to be TP53-dependent [208].

BBR has multiple intracellular targets such as nucleic acids and proteins involved in the regulation of cell growth including: DNA topoisomerase, estrogen receptors, NF-kappaB, TP53 and telomerase [209–213].

Some of the interactions of BBR and DNA and RNA may be due to the nitrogen atom at the 7-positon in the alkaloid BBR skeleton. This positively-charged nitrogen may result in the strong complex formations between BBR and nucleic acids and induce telomerase inhibition and topoisomerase poisoning [214, 215]. BBR have been shown to suppress BCL-2 and expression of other genes by interacting with the TATA-binding protein and the TATA-box in certain gene promoter regions [216, 217].

### Effects of berberine on induction of apoptosis, autophagy, cell cycle arrest and invasion

BBR has been demonstrated to have effects on the expression proteins involved in apoptosis, autophagy, cell cycle progression and invasion including: BCL-2, BCL-X_L_, PARP1, Beclin-1, TP53, p21^Cip1^, MMP9 and others [218]. Combined treatment with BBR and inhibitors of the Raf/MEK/ERK and PI3K/Akt pathways led to suppression of MMP-9 expression and invasion [219, 220].

### Effects of berberine on mitochondria and suppression of gero-conversion

BBR has been shown in some studies to localize to the mitochondria and inhibit the electron transport chain and activate AMPK. The effect of BBR on the premature stress-induced senescence that is induced by the chemotherapeutic drug mitoxantrone has been examined. BBR was determined to inhibit senescence by analysis of senescence-associated beta galactosidase, p21^Cip1^ induction, gamma H2AX expression and levels of ribosomal S6 protein phosphorylation. BBR was determined to alter gero-conversion from the process of cell cycle arrest to the induction of senescence. This was determined to be due to targeting the activity of mTOR/S6 and the generation of ROS [221, 222].

BBR has been shown to decrease mitochondrial membrane potential and intracellular ATP levels. BBR induces AMPK activation and inhibits mTORC1 phosphorylation by suppressing phosphorylation of S6K at Thr 389 and S6 at Ser 240/244. BBR also suppresses ERK activation in MIA-PaCa-2 cells in response to fetal bovine serum, insulin or neurotensin stimulation. Activation of AMPK is associated with inhibition of the PI3K/PTEN/Akt/mTORC1 and Raf/MEK/ERK pathways which are associated with cellular proliferation. The effects of low doses of BBR (300 nM) on MIA-PaCa-2 cells were determined to be dependent on AMPK as knockdown of the alpha1 and alpha2 catalytic subunits of AMPK prevented the inhibitory effects of BBR on mTORC1 and ERK activities and DNA synthesis. In contrast, higher doses of BBR inhibited mTORC1 and ERK activities and DNA synthesis by AMPK-independent mechanisms [223, 224].

### Effects of berberine on cancer and cancer associated pathways

BBR has been shown to have minimal effects on “normal cells” but has anti-proliferative effects on cancer cells (*e.g*., breast, liver, CRC cells) [225–227]. BBR induces G_1_ phase arrest in pancreatic cancer cells, while other drugs such as gemcitabine induce S-phase arrest [228].

### Berberine effects on bladder cancer

BBR was determined to enhance the effects of epirubicin (EPI) on T24 bladder cancer cells. EPI induced cell cycle arrest at G_0_/G_1_ and proteins associated with apoptosis and cell cycle arrest such as caspase 9 and cleaved caspase 3, BAX, TP53 and p21^Cip-1^. Co-treatment with BBR treatment resulted in enhanced levels of many of these proteins associated with apoptosis, increased ROS levels and decreased levels of BCL-2 [229].

### Berberine effects on brain cancer

BBR induces some of its anticancer effects by inducing apoptosis, autophagy, cell cycle arrest and cellular senescence. In some glioblastoma cells, BBR has been shown to inhibit EGFR signaling by suppression of the Raf/MEK/ERK pathway but not AKT signaling [230].

BBR can also induce autophagy in GBM. BBR targets the AMPK/mTOR/ULK1 pathway. This leads to autophagy flux which is accompanied by impaired glycolytic capacity. This results in reduced proliferative and invasive properties as well as increased apoptosis [231].

BBR has been shown to induce G_1_ arrest and apoptosis in glioblastoma cells by the mitochondrial/caspase pathway. The IC50 for BBR was determined to be 134 micrograms/ml. Increased p27^Kip1^ and decreased CDK2, CDK4, Cyclin D and Cyclin E were observed. Increased BAX/BCL2 ratio was observed. The mitochondrial membrane potential was disrupted and activated caspase 3 and caspases 9 were observed [232]. BBR can also inhibit arylamine N-acetyltransferase and DNA adduct formation in glioblastoma cells [233].

### Berberine effects on breast cancer

BBR can bind to the vasodilator stimulated protein (VASP). VASP is overexpressed in breast cancer cells with high mobility and inhibits actin polymerization. BBR binds VASP in MDA-MB-231 cells and suppressed proliferation and tumor growth [234].

BBR can also suppress the proliferation and migration of certain breast cancer cells by altering the activity of ephrin-B2 signaling. BBR treatment decreased VEGFR, Akt and ERK1,2 activation and the expression of MMP2 and MMP9 [235].

BBR was determined to suppress canine mammary gland tumors *in vitro*. These studied used BBR concentration ranging from 10-200 micromolar. Treatment with 10-200 micromolar BBR inhibited proliferation [236].

BBR has been shown to increase the anti-tumor effects of tamoxifen (TAM) in both drug-sensitive MCF-7 and drug-resistant MCF-7/TAM cells. The combined treatment of BBR and TAM resulted in enhance cytotoxic activity, G1 arrest and apoptosis potentially due to p21^Cip-1^ induction and increased BAX/BCL2 ratio [237].

The combination of BBR and CUR has been shown to be effective in suppressing the growth of certain breast cancer cell lines. The combined treatment was more effective than treatment with either BBR or CUR by themselves. The combined treatment resulted in phosphorylation of JNK and Beclin1 and decreased phosphorylation of BCL-2 [238].

BBR induced apoptosis by increased ROS in certain breast cancer cells (MCF-7 and MDA-MBA-231). JNK was activated by the treatment and triggered mitochondrial membrane depolarization. BAX was increased while anti-apoptotic family members such as BCL-2 were decreased [239].

BBR and tumor necrosis factor-related apoptosis-inducing ligand (TRAIL) have synergistic effects on inducing apoptosis in TNBC. p38^MAPK^ was activated in response to the combined treatment. Suppression of p38^MAPK^ enhanced BBR/TRAIL induced apoptosis [240].

The combination of BBR and cisplatin suppressed breast cancer growth by inducing DNA breaks and caspase 3-dependent apoptosis in breast cancer cells. Combining BBR and cisplatin resulted in potentiating the effects and BBR sensitized the cells to cisplatin. BBR increased the extent of DNA damage and apoptosis normally induced by cisplatin [241].

BBR decreased breast cancer cell migration and chemokine expression. This was determined by wound healing assays and RNA analysis of chemokine receptors in MCF-7 breast cancer cells [242].

Cotreatment of certain breast cancer cell lines with BBR and doxorubicin increased cytoxicity and apoptosis. BBR induced G_2_/M phase arrest in T47D but in G_0_/G_1_ in MCF-7 cells. The IC_50_ for BBR was determined to be approximately 25 micromolar after 48 hours of treatment for both cell lines [243].

BBR was determined to increase the activities of numerous proteins involved in proliferation including: JAK2, PI3K, AP-1 and NF-kappaB. These events lead to decreased IL-8 expression in the TNBC cell line MDA-MB-231. The IL-8 stimulated invasion and was suppressed by BBR. BBR also decreased MMP2, MMP9, E-cadherin, EGF, bFGF, and fibronectin in the breast cancer cells. The effect of BBR were inhibited by JNK and p38MAPK inhibitors but increased by p38^MAPK^ activators [244].

### Berberine effects on chemical carcinogen-induced cancers

BBR suppressed chemical carcinogen-induced tumors in mice and rats [245]. BBR (at high doses, 40 mg/kg) has been shown to suppress CRC in a chemical carcinogenesis model in mice. This inhibition was shown, in this study, to be through TOR inhibition and NF-kappaB activation [246].

### Berberine effects on cervical cancers

BBR increased TP53 mRNA transcripts in HeLa229 cells. In contrast BBR decreased BCL-2 and COX2 mRNA transcripts [247]. BBR has also been shown to have effects on HPV-18 E6-E7 oncoproteins by the targeting of TP53 in HeLa cells. This occurred by reprogramming of epigenetic modifications and altering the microtuble network. These studies suggest that BBR may be a promising agent for treatment of cervical cancer [248].

### Berberine effects on chondrosarcoma

BBR induced cell cycle arrest at G_2_/M in the HTB-94 chondrosarcoma cell line by increasing TP53 and p21^Cip1^ expression and decreasing cyclin B1, CDC2, CDC25c and pRB expression. BBR stimulated phosphorylation of both Akt and p38^MAPK^. Suppression of Akt and p38^MAPK^ signaling decreased the effects that BBR had on TP53 and p21^Cip1^ mediated cell cycle arrest and resulted in proliferation [249].

### Berberine effects on CRC

BBR inhibited intestinal polyps growth in the min/+ mouse model. This was shown to be due to regulation of macrophage polarity. Decreases in F4/80, mannose receptor (MR) and COX2 in the stroma and increases in iNOS occurred. BBR was shown to decrease the migration and invasion of the cells [250].

**Figure 11:**
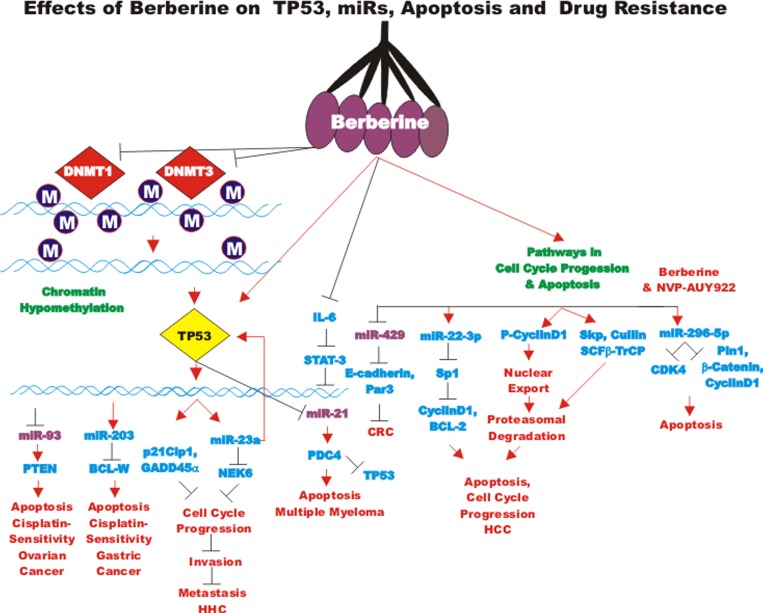
Effects of berberine on TP53, miRs, apoptosis and drug resistance Berberine can induce the expression of TP53 and miRs which can alter the expression of many genes involved in the regulation of apoptosis, cell cycle progression and drug resistance. miRs which are oncomirs are indicated in maroon, miRs which are tumor suppressor miRs are indicated in blue.

BBR suppressed the expression of E-cadherin and partitioning defective 3 (Par-3) in CRC. miR-429 was determined to be upregulated in CRC in comparison to normal tissues suggesting that miR-429 can function as an oncomir. Upon treatment with BBR and evodiamine (EVO) the levels of miR-429 in the tumor cells decreased [251]. A diagram depicting some of the effects of BBR on miRs. TP53 and regulation of cancer growth is presented in Figure 11.

BBR has been shown to synergize with the HSP-90 inhibitor NVP-AUY922 in inducing death of human CRC. A side effect of NVP-AUY922 treatment is the upregulation of survivin expression which can contribute to drug resistance. In contrast, when BBR was combined with NVP-AUY922, the growth of the normally resistant cells was reduced which was mediated by miR-296-5p-mediated suppression of the Pin1/beta-catenin/cyclinD1 signaling pathway and inhibition of CDK4 expression [252].

BBR inhibits COX2 and PEG2 in CRC. This results in decreased phosphorylation of JAK2 and STAT3 as well as MMP2 and MMP9 expression. BBR prevented the invasion and metastasis of CRC cells via inhibiting the COX2/PGE2 and JAK2/STAT3 signaling pathways. The JAK2 inhibitor AZD1480 blocked the effects of COX2/PGE2 on MMP2 and MMP9 expression. Thus, BBR suppressed CRC invasion and metastasis by inhibiting the COX2/PGE2 and JAK2/STAT3 signaling pathways [253].

### Berberine effects on endometrial cancer

BBR and MET will induce the lipolysis-stimulated lipoprotein receptor (LSR), which is a component of tricellular tight junctions in endometrial cancer cells. BBR and MET suppressed cell migration and invasion. This occurred by BBR and MET counteracting the effects of leptin on LSF expression [254].

### Berberine effects on esophageal cancer

Galangin is present in *Alphina officinarum Hance* and propolis (bee glue, a sealing mixture produced by honey bees). It has both anti-cancer and anti-oxidative properties. Combining galangin with BBR has been shown to have synergistic effects on inhibiting the growth of CRC. This combination resulted in increased ROS levels in esophageal cells. In contrast, treatment with either galangin or BBR by themselves suppressed WNT/beta-catenin signaling, the combination increased the suppression. The effects of these combination were also observed in in vitro and *in vivo* experiments [255].

BBR has been observed to inhibit the expression of the chemokine receptors (CXCR4 and CCR7) at the mRNA level in esophageal cancer cells. BBR suppressed the migration of KYSE-30 esophageal cancer cells [256].

### Berberine effects on gastric cancer

BBR has been shown to increase the activity of the EGFR inhibitors cetuximab and erlotinib against gastric cancer *in vitro* and *in vivo* [257]. The effects of BBR can be increased upon combination with d-limonene in gastric cancer cells. The combination of these two agents resulted in enhanced ROS production and apoptosis induction via the mitochondria-mediated intrinsic pathway and cell cycle arrest [258].

BBR has been shown to result in reduced IL-8 secretion in gastric cancer cells. This lead to decreased p38^MAPK^, ERK1,2 and JNK activities [259]. BBR treatment has been shown to reverse cisplatin-resistance and induce caspase-dependent apoptosis in two cisplatin-resistant gastric cancer cell lines. BBR induced miR-203 expression. Experimentally-induced miR-203 expression resulted in cisplatin-sensitivity. miR-203 bound the 3-UTR of BCL-W [260].

STAT3 and survivin signaling has been shown to be involved in chemotherapeutic drug resistance of gastric cancer. BBR and CUR will synergize with 5FU and target STAT-3 which can lead to sensitivity to 5FU in gastric cancer cells. However, in these studies, does of 29 micromolar were required for IC_50_ values [261].

### Berberine effects on HCC

BBR can stimulate G_2_/M cell cycle arrest in HCC and other cells. Hepatic nuclear factor 4 alpha (HNF4alpha), a key liver transcription factor, can transactivate the Exo 70 promoter region. BBR-mediated cell cycle arrest was determined to occur by down regulation of HNF4alpha and Exo-70 [262]. BBR has been shown to induce pyroptosis in HCC. Pyroptosis is a caspase-1 dependent programmed cell death program [263].

BBR can induce apoptosis and autophagic cell death in HEP-G2 HCC cells. Induction of apoptosis and autophagy were shown to require AMPK. This resulted in elevated expression of inactive acetyl-CoA carboxylase (ACC). Suppression of AMPK by RNAi or by the AMPK inhibitor (compound C) suppressed the effects of BBR. In contrast, the AMPK activator AICAR stimulated cytotoxic effects. BBR was shown to inhibit mTORC1 activation by stimulating AMPK [264].

miR-22-3p has been determined to be detected at decreased levels in HCC. BBR will increase miR-22-3p in HCC. In these studies, high doses of BBR (100 micromolar) inhibited cell growth at 24 hr. time interval. BBR treatment decreased the expression of SP1, cyclin D1 and BCL2. BBR induced miR-22-3p which bound SP1 and suppressed cyclinD1 and BCL2 [265].

BBR has also been shown to decrease cyclinD1 expression by inducing its proteasomal degradation in HEPG2 cells. BBR stimulates the Thr 286 phosphorylation of cyclin D1 which stimulates its nuclear export to the cytoplasm for proteasomal degradation. BBR also induced the component of the proteasome required for cyclin D1 proteasomal degradation, namely SKP, cullin and F-box containing complex-beta-transducin repeat containg protein (SCFbeta-TrCP) [266].

BBR has also been shown to induce plasminogen activator inhibitor-1 (PAI-1) and suppress uPA in HCC cells which suppressed their invasiveness and motility. BBR also induced COX2, NF-kappaB and MMP9 down regulation and inactivated MAPK and ERK1,2. The effects of BBR on uPA were attributed to the activation of PAI-1 [267].

BBR activates TP53 which increases the expression of miR-23a in HCC. BBR induced TP53 which in turn stimulated p21^Cip1^ and GADD45alpha expression. Suppression of miR-23a blocked binding of TP53 to the chromatin and blocked transcriptional activation of p21^Cip1^ and GADD45alpha. BBR induced miR-23a may suppress never in mitosis A (NIMA) kinase 6 (NEK6) and result in block of cell cycle in G_2_/M [268]. NEK6 may have effects on TP53. NEK6 has been shown to antagonize TP53 induced senescence [269]. By using RNA-Seq analysis, BBR has been shown to modify the expression of genes in the TP53 and cell cycle pathways [270].

BBR has been shown to increase the effectiveness of rapamycin in inducing the cell death of human hepatoma cells. The combination of BBR and rapamycin resulted in decreased levels of Thr 389-phosphorylated p70S6K compared to individual treatment [271].

The effects of BBR on the arachidonic acid (AA) metabolic pathway in HCC has been examined. BBR altered the viability and apoptosis of HCC cells in a dose-dependent fashion by inducing the translocation of apoptosis-inducing factor between the mitochondria and nucleus. BBR suppressed the levels of cytosolic phospholipase A2 (cPLA) and COX2 which increase the ratio of AA to PEG2 [272].

### Berberine effects on leukemia

BBR prepared from a *Berberis libanotica* (BI) extract was demonstrated to target NF-kappaB, COX2 and PI3K/Akt signaling to induce apoptosis by a mitochondrial/caspase dependent pathway in erythroleukemia cell lines [273].

### Berberine effects on lung cancer

The effects of combination of BBR with MEL have been examined in lung cancer cells. MEL was shown to enhance the effects of BBR on suppression of cell growth, colony formation and cell migration. MEL was demonstrated to promote cleavage of caspase 9 and PARP. MEL increased the effect of BBR on suppression of BCL-2 and increased apoptosis in part by stimulating the release of cytochrome C. MEL also promoted the effect of BBR on other biochemical events involved in cellular proliferation [274].

BBR has been shown to suppress STAT3 activity in human lung cancer cells. BBR inhibited doxorubicin-mediated STAT3 activation which was associated with sensitivity to doxorubicin treatment. BBR also suppressed the sphere forming capacity of these cells [275].

### Berberine effects on multiple myeloma

BBR has been shown to induce TP53 expression by suppressing the DNMT1 and DNMT3B DNA methyltransferases in MM which will result in TP53 gene expression as the methylation status of the TP53 promoter region changes [276]. This can alter TP53-mediated gene expression which includes many genes involved in regulation of the cell cycle as well as miRs including miR-21 which normally functions as an oncomir and can regulate the expression of PDCD4. miR-21 normally suppresses PDCD4. In the presence of BBR, miR-21 is suppressed. miR-21 is regulated by IL-6/STAT3 signaling which is also suppressed by BBR in MM cells [277].

BBR may affect multiple miRs in MM cells. The miR-99a~125b cluster was studied with t-anti-mirs (Antagomir) that have complete complementary antisense locked nucleic acids (LNAs) directed against mature miR-125b (anti-miR-125b). BBR was determined to suppress miR-99~125b, miR-17~92 and miR-106~25 in MM cells. The TP53, ERB and MAPK pathways were linked with the three miR clusters. In these studies, BBR suppressed the three miR clusters and downstream TP53, ERB and MAPK signaling pathways [278].

### Berberine effects on melanoma

BBR isolated from *Coptidis* rhizome, has been shown to inhibit the migration, EMT and invasion of melanoma cells. BBR suppressed PI3K/Akt and retinoic acid receptor (RAR) alpha signaling. In contrast, RARbeta and RARgamma signaling were upregulated. BBR treatment may reverse EMT in melanoma cells [279].

### Berberine effects on neuroblastoma

BBR has been shown to inhibit stemness, EMT and induce neuronal differentiation in neuroblastoma cells. BBR inhibited the expression of many genes associated with neuronal differentiation including: microtubule-associated protein 2 (MAP2), beta-III tubulin and neural cell adhesion molecule (NCAM) which resulted in neurons that were viable. In contrast, BBR also suppressed the expression of many genes associated with cancer stemness such as beta-catenin, CD133, NESTIN, N-MYC, NOTCH and SOX2. BBR induced cell cycle arrest at G_0_/G_1_ and suppressed proliferation which resulted in increased BAX/BCL2 ratio. BBR was shown to regulate EMT in part by downregulating the PI3K/Akt and RAF/MEK/ERK pathways and upregulating the p38^MAPK^ pathway [280].

### Berberine effects on oral cancer

BBR has been shown to have anti-cancer effects on KB oral cancer cells but not on the cell viability of normal primary human oral cells. BBR induced many markers associated with apoptosis such as DNA fragmentation, nuclear condensation and caspases 3 and 7. BBR induced the expression of FAS-L which resulted in activation of caspase 8, 9, 3 and PARP. In contrast, BBR treatment resulted in decreased BCL2 and BCL-X_L_ expression. Induction of FASL by BBR was determined to be dependent on p38^MAPK^ which resulted in decreased MMP2 and MMP9 expression [281].

### Berberine effects on osteosarcoma

BBR has been shown to suppress proliferation and induce DNA damage in osteosarcoma MG-63 cells [282]. BBR was also shown to inhibit proliferation, induce apoptosis and suppress PI3K/Akt signaling in U20S human osteosarcoma cells. BBR treatment resulted in increased BAX and PARP expression and decreased BCL2 expression [283].

### Berberine effects on ovarian cancer

BBR suppressed proliferation of SKOV3 ovarian cancer cells. BBR decreased the expression of BCL-2 and survivin while increasing the expression of BAX. BBR was demonstrated to synergize with cisplatin in inducing death of SKOV3 cells. BBR treatment resulted in restoration of the demethylation status of MutL homolog 1, colon cancer, nonpolyposis type 2 (hMLH1) promoter region and increased its expression. hMLH1 encodes a protein involved in DNA mismatch repair [284].

BBR has been shown to augment the effects of cisplatin in inducing cell cycle arrest in A2780 ovarian carcinoma cells. BBR inhibited miR-93 expression and increase the expression of the PTEN tumor suppressor which is a target of miR-93. miR-93 levels were higher in cisplatin resistant A2780 cells [285].

### Berberine effects on pancreatic cancer

In MIA-PaCa-2 cells, the *KRAS* gene is mutated and results in the activation of the PI3K/PTEN/Akt/mTORC1 and Raf/MEK/ERK pathways. Both pathways are usually associate with proliferation. MIA-PaCa-2 cells also lack WT TP53, which can have effects on cell cycle progression, proliferation, cellular senescence and tumorigenicity. BBR treatment was determined to inhibit DNA synthesis and proliferation of the pancreatic cancer cells. So, in these studies, the growth inhibitory effects of BBR were independent of WT TP53. MET was determined to induce similar effects in the pancreatic cancer cells. In addition, tumor xenograft experiments were performed. MIA-PaCa-2 cells were implanted into the right flanks of *nu/nu* mice. When the tumors reached a mean diameter of 2 mm, the animal were treated with BBR (5 mg/Kg), MET (250 mg/Kg) or vehicle control. The drugs were administered once daily intraperitoneally (50 microliters/mouse). This protocol resulted in a decrease in the size of the xenografted tumors by 70% at the end of the experimental protocol (day 29). MET treatment also reduced tumor volumes by similar amounts. In this study, BBR inhibited DNA synthesis, cell cycle progression, proliferation *in vitro* and tumor growth in mice. BBR was also observed to be well tolerated and did not affect the weight of the mice [223, 224].

BBR has been shown to suppress genes associated with stemness such as SOX2, POU5F1, NANOG and NOTCH in the side population of certain pancreatic cancer cell line PANC-1. The side population fraction is frequently associated with CSCs [286].

### Berberine effects on prostate cancer

In certain prostate cancer cell lines, BBR induced ROS production which resulted in TP53 translocation to the mitochondria. When TP53 was present in the mitochondria, it interacted with cyclophilin D (Cyp D) which resulted in the opening of the mitochondrial permeability transporter (mPTP) [287].

BBR has been shown to decrease 22Rv-1 prostate cancer growth and cellular testosterone formation. These studies have indicated that BBR may be appropriate as a skeleton backbone for the design of aldo-keto-reductase family member inhibitors (AKR1C3) [288].

BBR treatment results in suppression of migration and invasion of prostate cell cells. BBR treatment resulted in decreased expression of certain mesenchymal genes such as: bone morphogenetic protein 7 (BMP7), nodal growth differentiation factor (NODAL) and SNAIL. The expression of these genes is normally associated with shorter survival of prostate cancer patients [289].

BBR inhibited HIF-1alpha and VEGF expression in prostate cancer cells and increased their radio-sensitivity in *in vitro* as well as in animal studies [290].

BBR can target EGFR signaling to suppress the proliferation of prostate cancer cells *in vitro*. BBR was shown to accumulate inside the cells that were in G_1_ phase and increased the induction of apoptosis. BBR suppressed both prostate specific antigen expression and EGFR activation after EGF treatment [291].

### Berberine effects on skin squamous cell carcinoma

BBR has been shown to inhibit the proliferation of squamous skin cell carcinoma cells. BBR was demonstrated to induce apoptosis and inhibit migration of the skin cancer cells [292].

### Berberine effects on thyroid carcinoma

BBR has been shown to inhibit the activity of the RET tyrosine in medullary thyroid carcinomas (MTC). This occurred by stabilization of G-quadruplex structures present on the RET promoter region in the MTC TT cell line. In contrast, minimal effects were observed on the papillary thyroid TPC1 cell line. The TPC1 cell line has a chromosomal rearrangement and they lack the G-quadruplex forming region in the RET promoter region [293].

### Berberine effects on uterine leiomyoma

BBR inhibited proliferation of human uterine leiomyoma (UtLM). The expression of cyclin A1, cyclin B, and Cdk1 were decreased upon BBR treatment, while the expression of Cdk4, p21^Cip1^ and TP53 were increased. BBR induced upregulation of BAX and Annexin V staining. In contrast, BBR did not appear to affect cell proliferation in “normal” human uterine smooth muscle (UtSMC) cells [294].

### Berberine effects on TP53 signaling

Activation of the TP53 pathway has been reported after BBR treatment. However, we and others have demonstrated that BBR and modified derivatives can exert effects in cells containing mutant *TP53*. Some authors have claimed that the effects of BBR in some cancer cells are TP53-dependent. The effects of BBRs on neuroblastoma cells have been postulated to be TP53-dependent [295]. However, the concentrations of BBR used in these studies were often very high from 25 to 100 micromolar and often the concentrations of glucose were high (25 mM). The effects of BBR and MET on pancreatic cancer cells have been determined to be affected by glucose concentrations as low glucose concentrations (3 mM) result in AMPK-dependent effects while high glucose concentration result in AMPK-independent effects. Some studies have also been performed with prostate and lung cancer cells. However, in some of these studies, different cell lines were used as well as culture conditions to make these conclusions and the cells may have additional mutations besides *TP53* which make them sensitive or resistant to BBRs. The IC_50_s for BBRs were over 50 micromolar. These studies examined the effects of BBR on prostate and lung cancer tumors in mice respectively [296, 297].

### Effects of berberine on bacteria

BBR has been shown to have anti-bacterial effects by increased ATP-induced inflammasome activation. BBR treatment resulted in macrophages releasing more caspase-1 p10 and IL-1beta. Suppression of AMPK prevented these effects. BBR also stimulated macrophages to kill engulfed bacteria and increased the survival of mice injected with certain bacteria [298].

BBR has been shown to inhibit *Fusobacterium nucleatum* and associated opportunistic pathogens during CRC progression. BBR is believed to modulate the colonic tumor microenvironment [299].

BBR and other components of essential oils (cinnamaldehyde eugenol and thymol), have been shown to have some effects when combined with streptomycin on food-borne bacteria such as *Listeria monocytogenes* and *Salmonella Typhimurium* [300].

### Modified berberines

Some derivatives of BBR have been made by cyclizing BBR. A35 targets topisomerase 1 (TOP1) and TOP2alpha. A35 can intercalate DNA and promote top2alpha cleavage complex formation. Thus, A35 is a topoisomerase poison. Studies with mice indicated that no cardiotoxicity was observed at least at the A35 concentrations examined [301].

The BBR-8 compound (Ber8) is a 9-substituted BBR. Ber8 has been shown to induce cell growth arrest and senescence in cancer cells. Ber8 induced DNA damage at the telomeric regions of the chromosome. Ber8 stabilized G-quadraplexes at the telomere. Ber8 stimulated the telomeric repeat factor 1 (TRF1) and protection of telomeres 1 (POT1) proteins and displaced them from the telomeres. This resulted in telomere uncapping [302].

Novel phenyl-substituted BBRs have been created by copper-catalyzed azide-alkyne cyclo addition (click chemistry). Some of the compounds exhibited activity against MCF-7 breast cancer cells, SW-1990 pancreatic cancer cells and SMMC-7721 liver cancer cells. In contrast, less effects were observed with noncancerous human umbilical vein endothelial cell (HUVEC) cell lines [303, 304].

Other BBR derivatives have been synthesized. BBR compounds containing a single and four phenolic groups exhibited increased up-regulation of superoxide dismutase (SOD) gene expression. The effects of the compounds were examined on human fibrosarcoma cells (HT1080). Some of the modified BBRs were determined to have enhanced activities [305].

BBR dimers have been synthesized which display high affinity in binding G-quadruplexes [306].

**Figure 12:**
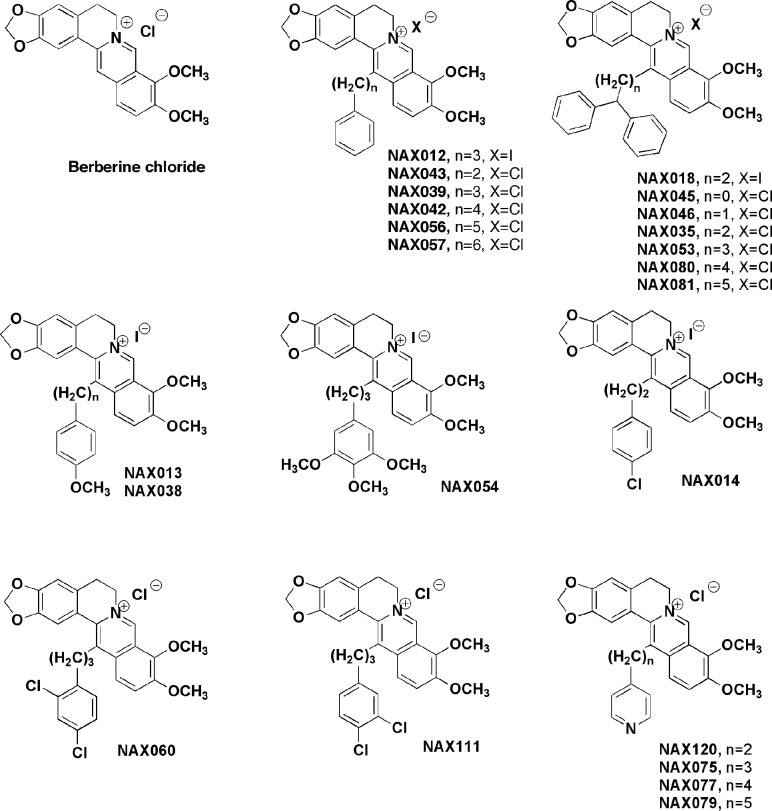
Chemical structures of modified berberines from naxospharma

A panel of modified BBRs has been developed by Naxospharma in Milan, Italy [307, 308]. The structures of the modified BBRs are presented in Figure 12. A key feature of the modified BBRs is the presence of (hetero)aromatic moieties which have been added to the 13-position of BBR alkaloid skeleton. This was achieved by addition of a hydrocarbon linker of variable length and functionality. These modifications were made to generate modified BBRs that may have a geometric propensity for additional stacking-type, noncovalent interactions that might interact with their intracellular targets in a more effective fashion resulting in enhanced biological and anti-cancer effects [309–314].

The synthesis of these compounds has been described. The purity of this compound has been ascertained by HPLC on a Jasco system LC-2000 series with an Agilent Eclipse XDB-C18 (4.6 mm3 150 mm3 3.5 mm) column with a flow rate of the mobile phase (50% water, 50% acetonitrile plus 0.1% trifluoroacetic acid) that was maintained at 1.0 mL per minute. Absorbance was measured at 235, 265, 340, and 420 nm. The modified BBR NAX014 was less toxic than the non-modified BBR as 30.9 mg/kg and 10.9 mg/kg were required for the lethal dose (LD_50_s) respectively [312].

When the spacer between the 13 position of the BBR backbone and the added phenyl ring(s) was longer than 3 C-atoms in length, increased cytotoxicity was observed. The LD_50_ for mice treated with BBR and some modified BBRs has been examined. BBR and modified BBRs induced apoptosis and cellular senescence and reduced microvesicle density in a HER2-overexpressing transgenic mouse model. The modified BBR NAX014, in this study, has been demonstrated to suppress (delay) tumor formation better than unmodified BBR at a “safe-dose” of 2.5 mg/kg twice a week. NAX14 induced growth inhibition and tumor vascularization through its anti-angiogenic effect. Some modified BBRs have been reported to be active on cells with either WT or mutant TP53 [308–314].

### Nanoencapulated berberines

BBR has been encapsulated in liposomes. This has been performed due to the poor aqueous solubility and rapid metabolism and to improve gastrointestinal absorption. The effects of BBR in nanoparticles have been examined in some cancer cell lines such as MCF-7, HepG2 and A549 [315].

BBR solid lipid nanoparticles (SLN) were observed to improve bioavailability. BBR-SLNs were determined to suppress weigh gain, fasting blood glucose levels and to improve insulin tolerance in *db/db* diabetic mice. They also improved lipid function [316].

The effects of BBR in nanoparticles have also been examined on hepatosteatosis. The effects of BBR SLN in nanoparticles on lipid metabolism in the liver were examined in *db/db* diabetic mice. The BBR SLNs inhibited body and liver weight gain. Serum alanine transaminase and liver triglycerides were also reduced in the mice. The highest levels of the drug were present in the liver. The BBR SLNs reduced the levels of fat accumulation. BBR SLNs also decrease the expression of key lipogenic genes. These results suggest that BBR SLNs may be eventually used for treatment of hepatosteatosis [317].

Specialized BBR ferrous magnetic mesosporous Janus nanoparticle, (Fe3O4-mSiO2 NPs) have been developed to target liver cancers. These specialized NPs have a pH-sensitive group on the surface of the mesoporus SiO2 to creat tumor microenvironmental nanoparticles that display sensitivity for HCC cells [318].

A phospholipid complex consisting of berberine in cucurbitacin B modified was made to try to increase the bile duct targeting deliver to cholangiocarcinoma (CC) [319].

BBR liposomes have been synthesized to treat certain drug-resistant breast cancer including CSCs. The BBR liposomes were demonstrated to cross the CSC membrane and inhibit drug transporters including Abcc1, ABCC2, ABCC3 and ABCG2. The BBR liposomes accumulated in the mitochondria and resulted in the activation of BAX and inhibition of inhibition of BCL2. This resulted in the induction of apoptosis [320].

### Compounds derived from similar plant sources

*Berberis libanotica Ehrenb* (BLE) is a plant rich in alkaloids. Treatment of the prostate cancer cell lines (DU145, PC3 and Rv22-1) with BLE inhibited their growth and induced cell cycle arrest in G_0_/G_1_. BLE also suppressed spheroid forming capacity [321]. The related drug Berbamine, which is sometimes derived from the same plants, inhibits HCC tumor growth in tumor transplant studies [322].

### Effects of activators of AMPK on cancer

AMPK is a critical kinase involved in monitoring the energy status in cells. Hence it plays critical roles in cell growth, cell cycle progression, protein synthesis, survival and tumorigenesis. AMPK has been shown to have effects on CSCs. The anti-diabetes drug MET is known to activate AMPK. Related drugs of the biguanide class of antidiabetic drugs which lower blood sugar levels were initially discovered in plants (French lilac or goat’s rue *Galega officinalis*). There are approximately 272 clinical trials examining the effects of MET and cancer. In addition, natural compounds in our diet such as: capsaicin (present in chili peppers), genistein (present in soybeans and other plants), epigallocatechin gallate (EGCG, present in green tea) and quercetin (present in many fruits, berries and vegetables and especially in capers) will also activate AMPK. There are approximately 8 and 13 clinical trials examining the effects of capsaicin or quercetin on cancer respectively. Novel EGCG analogs have been recently determined to active AMPK more efficiently than MET. These compounds were examined on breast cancer cells with stem like properties. The novel EGCG compounds induced p21^Cip1^ and decreased activity of the PI3K/PTEN/Akt/mTORC1 pathway in the breast cancer cells with stem like properties [323].

Interestingly, BBR and MET may have some similar properties and both have been used to treat type II diabetes in traditional medicine (BBR) and in clinically (MET). MET treatment decreased in blood glucose levels by reducing hepatic gluconeogenesis and elevating glucose uptake. MET activates AMPK which suppresses mTORC1 activity. mTORC1 activity is frequently elevated in CSC including pancreatic CSCs [223, 224, 324].

MET may induce S phase arrest in certain pancreatic cancer cell lines [324]. MET may target pancreatic CSC more than non-CSCs. MET has been shown to increase ROS in the CSC population which resulted in reduced mitochondrial transmembrane potential. This resulted in a lethal energy crisis and was AMPK/mTORC1 independent. While MET treatment of the CSCs blocked their ability to expand *in vitro* and induced apoptosis, MET treatment of the non-CSCs induced cell cycle arrest but they were not eliminated [325.326]. More recent studies have shown that MET may induce G_1_ phase arrest via suppression of miRs such as miR-221 in pancreatic cell lines such as PANC1. This resulted in increased sensitivity to TRAIL through upregulation of death receptor 5 (DR5) [327]. MET treatment resulted in down-regulation of miR-221 and increased expression of the cell cycle inhibitor p27^Kip-1^. miR-221 is normally involved in tumor development and can regulate TRAIL resistance. While treatment of pancreatic cancer cell lines with MET or TRAIL by themselves did not appear to induce significant apoptosis as judged by the percentage of Sub-G_1_ population detected by propidium iodine staining and flow cytometric cell cycle analysis, the combined MET and trail treatment increased the Sub-G_1_ population in all three pancreatic cancer cells examined which was due to the interaction between caspases, TRAIL and DR5. In these studies, the effects of MET on miR-221 expression may be important in G_1_ phase arrest but not apoptosis. An inverse relationship between miR-221 and MET treatment has been observed in type 2 diabetes patients [328].

Diabetes patients treated with MET may have a reduced risk of pancreatic and other cancers [329–331]. There are/have been at least sixteen clinical trials examining the effects of combining MET with various drugs used in the treatment of pancreatic cancer.

### Effects of metformin on liver diseases

MET can also inhibit pathways critical for hepatic lipogenesis. Inhibition of these pathways by MET can suppress liver tumorigenesis [332]. There have been at least five clinical trials with metformin and liver cancer. MET can activate AMPK and SIRT1 expression and alleviate hepatosteatosis [333].

### Other plant-derived chemicals on cancer

*Momordica charantia* is commonly known as: bitter melon, bitter gourd, and balsam apple. It is cultivated world-wide and both the plant and the fruit are consumed. Bitter melon has been evaluated in at least three clinical trials with diabetes and metabolic syndrome patients. It has been shown to have many important medical properties including: anti-bacterial, anti-cancer, anti-diabetic, anti-inflammatory, anti-obesity, anti-oxidant and recently it has been postulated to target CSCs. The bitter melon plant contains several components which can have effects on suppression of cell growth such as: cucurbitane type triterpenoids, essential oils, fatty acids, flavonoids, phenolic acids, proteins, triterpene glycosides and saponins [334].

Recent studies have determined that bitter melon extracts contain compounds that inhibit growth of many types of cancer and ameliorate resistance to common chemotherapeutic compounds. Bittermelon extracts can inhibit ovarian cancer growth via activation of AMPK and suppression of the mTOR/p70S6K and/or the AKT/ERK/FOXM1 (Forhead Box M1) signaling cascade [335]. Alpha-Momorcharin, a ribosome inactivating protein, present in bitter melon extracts may in part be responsible for the anti-cancer effects [336, 337].

Many diverse natural products have been examined for their effects on the SHH pathway. SHH signaling has been strongly implicated in cancer development, CSCs, progression and resistance. The effects of various natural products such as: CUR, cyclopamine (a teratogen isolated from the corn lily *Veratrum californicum*), EGCG, genistein (a phytoestrogen isoflavone present in soy and other beans), norcantharidin (a derivative of cantharidin obtained from the blister beetle (*Mylabris phalerata Pallas*), RES, zerumbone (a ginger plant from Southeast Asia) and even arsenic trioxide have been examined on SHH signaling and summarized recently [338]. EGCG has been evaluated in at least 2 clinical trials with various types of cancer patients including gastric and prostate cancer. There are at least 33 clinical trials with genistein and various cancer patients.

The effects of sulforaphane (present in broccoli, brussel sprouts, cabbage and other cruciferous vegetables), quercetin (a flavonol found in many fruits, grains, leaves and vegetables) and catechins have been examined on let-7 miR induction and KRAS suppression in pancreatic cancer cells. Combining green tea catechins (GTCs) with sulforaphane or quercetin was observed to have enhanced effects on pancreatic cancer cells as measured by suppression MMP-2, MMP-9 and ALDH1 activities, colony and spheroid formation and the induction of apoptosis. These treatments resulted in induction of the let7 miR and suppression of KRAS [339]

Certain African potatoes are sources of the hypoxoside derivative Rooperol. Rooperol was determined to inhibit the proliferation of OCT4-expressing human embryonal carcinoma NT2/D1 cells which have some properties of CSCs. Rooperol induced the production of ROS and induced cell death via altering the mitochondrial membrane potential. Interestingly, in these studies, the effects of rooperol were determined to be TP53-dependent and rooperol induced TP53 activation and apoptosis. Rooperol treatment decreased OCT4 expression and other genes associated with stemness such as NANOG and SOX2 [340].

Pomiferin is derived from the fruit of *Maclura pomifera* (a.k.a Osage orange). This fruit has been used by various cultures for medicinal purposes. Pomiferin has been shown to inhibit the growth, self-renewal and invasion of glioma neuorspheres. Pomiferin inhibited the expression of many genes associated with stemness including: BIM1, NANOG and NESTIN [341].

Extract prepared from *Iberis amara* (a.k.a. Bitter candytuft) have been shown to inhibit CRC in both *in vitro* experiments and in the HT-29 tumor xenograft model. Some of the anti-tumoral effects of the *Iberis amara* extract were determined to occur by the induction of apoptosis and ROS [342].

Parthenolide is derived from the plant feverfew. The effects of the natural products parthenolide and andrographolide have been examined on MM CSCs. NF-kappaB is a target of both parthenolide and andrographolide. Both parthenolide and andrographolide exhibited toxicity towards the MM-CSCs as opposed to the non-tumorigenic MM cells [343].

Certain natural products (*e.g*., CUR, genistein, and quercetin) can serve as radiosensitizers and may be more effective than synthetic compounds in killing radioresistant CSCs after radiotherapy. miRs are important in the regulation of radiation resistance. Certain natural products may alter (induce) the expression of miRs that inhibit genes which are involved in resistance and tumor progression [344].

Beta-carotene (present in carrots and other fruits and vegetables), CUR, EGCG, piperine (present in black pepper), sulforaphane, genistein all have been shown to target and kill CSCs. There are approximately 16, 57 and 20 clinical trials examining the effects of beta-carotene, curcumin or sulforaphane on cancer. These phytochemical exert part of their growth suppressive effects via inhibition of the WNT signaling pathway which plays key roles in CSCs [345].

### Effects of Omega-3 polyunsaturated fatty acids on cancer

There are many sources of omega-3 poly unsaturated fatty acids (PUFA) including cold water fish and flax seed and flax seed oil. Fish oil is composed of two types of omega-3 PUFA, icosapentaenoic acid (EPA) and docosahexaenoic acid (DHA). Flax seed oil contains alpha-linolenic acid (ALA) which is present in flax seeds, hemp, walnuts and pumpkin seeds. The top eleven fish in terms of omega-3 PUFA are: mackerel, lake trout, herring, Bluefin tuna, salmon, canned sardines, Atlantic sturgeon, albacore tuna, lake whitefish, Anchovies and bluefish. Other fish contain less omega-3. PUFA. PUFAs have been shown to suppress the growth of certain primary tumors. Recently the effects of PUFAs on recurrent CRCs have been examined in the presence and absence of FOLFOX chemotherapy. The effects of the PUFA on FOLFOX-resistant CRC CSCs were also determined. These studies observed that the effects of PUFA could be enhanced by FOLFOX chemotherapy. The combination was more potent in inhibiting various aspects of CRC CSC biology. The combination treatment resulted in increased apoptosis and PARP cleavage and suppressed PI3K/PTEN/Akt/mTORC1 signaling [346]. There are approximately 147 clinical trials examining the effects of omega-3 PUFA and cancer.

## SUMMURY

The beneficial effects of natural products/nutraceuticals are being examined for their effects on human physiology in many diverse settings, from: aging, cancer, cardiovascular diseases, joint inflammation and neurodegenerative diseases. Clearly, more attention and research should be devoted to research on the beneficial and potential detrimental effects of nutraceuticals. Many of the effects of natural products/nutraceuticals are mediated by miRs and the induction of AMPK and ROS or suppression of the WNT/beta-catenin, PI3K/Akt/mTOR and RAS/MEK/ERK signaling pathways. Some studies have indicated that high concentrations of various neutraceuticals may be required to elicit the desired effects. However, a good, well-balanced diet may prevent certain diseases from occurring. Development of more effective delivery methods and chemical modifications which result in more potent compounds with better PD/PK properties may improve the nutraceuticals for the treatment of certain diseases. Furthermore, more emphasis needs to be dedicated to educating our youth on the benefits of natural products/nutraceuticals and the potential harm of a high fat, sedentary life style.
